# Accommodative Behavior of Non-porous Molecular crystal at Solid-Gas and Solid-Liquid Interface

**DOI:** 10.1038/srep14460

**Published:** 2015-09-28

**Authors:** Hemant M. Mande, Prasanna S. Ghalsasi

**Affiliations:** 1 Department of Chemistry, Faculty of Science, The Maharaja Sayajirao University of Baroda, Vadodara-390002, Gujarat, India

## Abstract

Molecular crystals demonstrate drastically different behavior in solid and liquid state, mainly due to their difference in structural frameworks. Therefore, designing of unique structured molecular compound which can work at both these interfaces has been a challenge. Here, we present remarkable ‘molecular’ property by non-porous molecular solid crystal, dinuclear copper complex (C_6_H_5_CH(X)NH_2_)_2_CuCl_2_, to reversibly ‘adsorb’ HCl gas at solid-gas interface as well as ‘accommodate’ azide anion at solid-liquid interface with crystal to crystal transformation. The latter process is driven by molecular recognition, self-assembly, and anchimeric assistance. The observed transformations are feasible due to breathing of inner and outer coordination sphere around metal center resulting in change in metal polyhedra for ‘accommodating’ guest molecule. These transformations cause changes in optical, magnetic, and/or ferroelectric property offering diversity in ‘sensing’ application. With the proposed underlying principles in these exceptional reversible and cyclic transformations, we prepared a series of compounds, can facilitate designing of novel multifunctional molecular materials.

Designing of a smart material capable of operating at maximum possible interfaces which can display swift and prominent change of its ‘measurable property’ due to external chemical or physical stimuli has been of keen interest in science^1^,^2^. The field has been of profound importance related to technology, environmental safety and energy issues. This direction has driven research in coordination polymers or metal-organic framework (MOF) compounds to form containers to porous compounds in last two decades^3^,^4^,^5^,^6^. Typically, these types of compounds act as a ‘sensor’ material by efficiently adsorbing-desorbing various guest (sensed) molecules in their ‘porous’ spaces with the use of non-covalent interactions. Ideally a ‘sensor’ compound during adsorption-desorption must show (a) change in colour (optical property); (b) change in symmetry (polar space group to non-polar space group or *vice-versa*); (c) selectivity; (d) complete reversibility; and/or (e) retention of crystallinity.

Most of these sensors work at solid-gas interface by adsorption-desorption of gases, and at solid-liquid interface by ‘molecular recognition’ of ions^7^,^8^,^9^,^10^. But till date, to our best knowledge, no sensor has shown flexibility to work at both solid-gas as well as solid-liquid interface. This may be due to complete difference in ‘designing principles’ and ‘mechanisms’ involved at these interfaces. The major dissimilarity with respect to structural design remains in the geometry of the central metal ion and the type of organic ligands employed. For solid-gas interface, ‘square planar with octahedral geometry’ of the metal ion is preferred with carboxylic acid or secondary amines as organic ligands for generating rigid porous^11^ or non-porous structures^12^,^13^. This is in contrast to flexible network of mainly ‘tetrahedral with octahedral structures’ with metal-ether, or metal-primary amine linkages for solid-liquid or molecular recognition purpose^13^. We mixed these two structural designing aspects, by employing only square planar geometry around central metal center with primary amines as ligands, to form a unique chemical sensor which operates at solid-gas and solid-liquid interface. This sensor offers both rigidity and structural reversibility due to square planar structure and molecular recognition/dynamism due to primary amines. This also helped in revealing chemistry behind the crystal to crystal transformation observed during our present work. But, to our best knowledge, square planar-primary amine based structures are very rare. Concurrently, we synthesized series of non-porous molecules, by the reaction of derivatives of benzylamine and CuCl_2_, with similar structural framework. Here, we will discuss reversible adsorption-desorption of HCl gas at solid-gas interface, and selective accommodation of N_3_^−^ anion at solid-liquid interface with crystal to crystal transformation, representatively shown for one compound in Fig. 1.

An ‘accommodative’ behavior is new a concept to design not only sensors but also for multifunctional or multiferroic compounds. In these type of compounds ‘breathing’ nature of inner and outer coordination sphere around metal ion forces change in geometry and drive self assembly in solid state, akin to a archetypal molecular property in liquid state.

## Results and Discussion

### Crystal to Crystal transformation at Solid-Gas interface: Designing strategy

During thermal investigation of organic-inorganic hybrid materials, A_2_CuCl_4_ (where A = derivatives of aniline, benzylamine, aliphatic amines), most of the compounds in series, loose organic amine prior to the liberation of HCl/Cl_2_ gas, except exactly opposite behavior for the derivatives of benzylamine^14^. This result suggests a stable Cu(II)-benzylamine (and its derivatives) complex which desorb HCl gas and hence ‘might re-adsorb’. To observe this latter possibility we prepared (C_6_H_5_CH_2_NH_2_)_2_CuCl_2_ complex (**A_1_**)^15^. Single crystal X-ray study revealed that this compound has 1-D chain structure due to two equatorial Cl-Cu-Cl bridging in a trigonal pyramidal Cu(II) center (see Supplementary Fig. S1(a) online). When these crystals were subjected to dry HCl gas changes colour completely. The molecular structure of the newly formed polycrystalline compound was accomplished indirectly by synthesizing the same compound (as single crystals) by a direct reaction between CuCl_2_ and benzilinium chloride in aqueous medium (see Supplementary Scheme S1 and Fig. S1(b) online). The powder-X ray pattern of the final compound matched perfectly with the powder-x-ray data simulated from the single crystal data on A_2_CuCl_4_, (**B_1_**) (see Supplementary Fig. S2 online). **B_1_** crystallizes in nonpolar space group, where CuCl_4_^2−^ forms a 2-D layered structure which is flanked by benzylammonium cation on both the sides. We observed similar layered structure for other compounds, (*p*-ClC_6_H_5_CH_2_NH_3_)_2_CuCl_4_ (B_2_)^16^ and (C_6_H_5_CH(CH_3_)NH_3_)_2_CuCl_4_(B_*5S*_).

Coordination complex **A_1_** is stable under ambient conditions and shows change in its inner and outer coordination sphere, (see Supplementary Table S3 online) after exposure to an atmosphere of hydrochloric acid vapor. Due to lack of pores, interestingly, this process happens with movement of atoms in the solid state with the retention of crystallinity, crystal to crystal transformation. Although similar observations are made in literature, exact mechanism for the same remained elusive^17^.

### Crystal to Crystal transformation Solid-Gas interface: Probing Mechanism

The chemisorption of HCl occurs with dramatic colour change from dark green (**A_1_**) to yellow (**B_1_**) from over 30 minutes up to 150 minutes in 100% yield, as demonstrated unequivocally by monitoring using FT-IR (Fig. 2) and XRPD (Fig. 3). We observed this transformation happens in two main steps, as shown mechanistically in Fig. 4. In the first step due to adsorption of HCl gas benzylamine changes its role from ligand (inner coordination sphere) to counter cation with the formation of benzylammonium cation at the cost of breaking all covalent Cu−N and H−Cl bonds (Fig. 4b). This is observed clearly in FT-IR by gradual decrease in intensity of vibrations of amine bonds and formation of ammonium moiety. Powder x-ray also reveals the changes occurred in the planes where (benzylamine) C-N atoms are located (clearly shown in tabulated form: (see Supplementary Table S3 online). This process gets completed in 60 minutes (rate of reaction can be controlled by the concentrations of reactants and the flow of HCl gas). This process is re-evident because the addition of base (KOH) inserts back benzylamine in inner coordination sphere from counter cation position, by abstracting proton, as cleanly seen in FT-IR and powder x-ray (see Supplementary Fig. S3 online). The second step, is most crucial, where accommodating ability of Cu(II) is observed due to change in its hybridization from (d^2^)sp^2^ to dsp^2^ with a cross-dimeric eight (8) membered ring formation (analogues to classical example of AlCl_3_ to Al_2_Cl_6_) where bonds are formed between Cu(II) and neighboring unbound -Cl (p-orbital) atom, attached to another Cu(II) atom in a 1-D chain (Fig. 4c,d). This means charge neutrality is achieved by 1-D 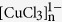 chain by extending its growth at the chain boundary interface to form a layered structure with nearly perfect squares of [Cu_4_Cl_4_]_n_ units. Interestingly, inversion center is observed exactly between these 1-D layers on {028} plane (2*θ* = 26*°*), (shown by orange colour in Fig. 4c) which diminish during the process of adsorption.

We would like to point out that (a) A_1_ is not soluble in water, therefore role of water, micro crystallization is ruled out; (b) the rate of inter-conversion between trigonal bipyramidal and square pyramidal structures in liquid state is very fast, because of negligible energy difference between these structures.

### Accommodative nature at Solid-Gas interface: Stereochemistry of Crystal to Crystal transformation

The above mechanism is not able underline stereo-chemical changes during the transformation. This directed us to synthesize (see Supplementary Scheme S2 online) a pair of enantiomerically pure complexes ((*R*)-(+)-*α*-ethyl phenyl amine)_2_CuCl_2_ (**A**_***R***_) and ((*S*)-(−)-*α*-ethyl phenyl amine)_2_CuCl_2_ (**A**_***S***_))^18^ and their respective double salts ((*R*)-(+)-*α*-ethyl phenyl ammonium)_2_CuCl_4_ (**B**_***R***_) and ((*S*)-(−)-*α*-ethyl phenyl ammonium)_2_CuCl_4_ (**B**_***S***_)), using *α*-ethyl phenyl amine as a chiral ligand. Single crystal x-ray revealed that pair of **A**_***R***_-**A**_***S***_ and **B**_***R***_-**B**_***S***_ have similar structure but are not having 1D chain (similar to A_1_) or 2D layered structure (similar to B_1_). **A**_***R***_ crystallizes in monoclinic chiral space group *P*1 at RT (see Supplementary Fig. S5 online). A dinuclear complex due to long sheared Cu-Cl bond between two slightly distorted square planar Cu(II) centers. No prominent hydrogen bonding and porosity was observed in crystal packing. **B**_***R***_ crystallizes in monoclinic chiral space group *C*_2_ at RT (see Supplementary Fig. S6 online) with isolated highly distorted tetrahedral geometry around CuCl_4_^2−^ anions (Cl-Cu-Cl *trans* angle 152.21°) with three N−H···Cl (3.180(2) Å) hydrogen bond and two bifurcated 2(N−H)···Cl (3.228(3) and 3.235(1)Å) hydrogen bonds. The data summarizing crystal structure and refinement parameters are listed in Supplementary Table S4.

**Figure 1:**
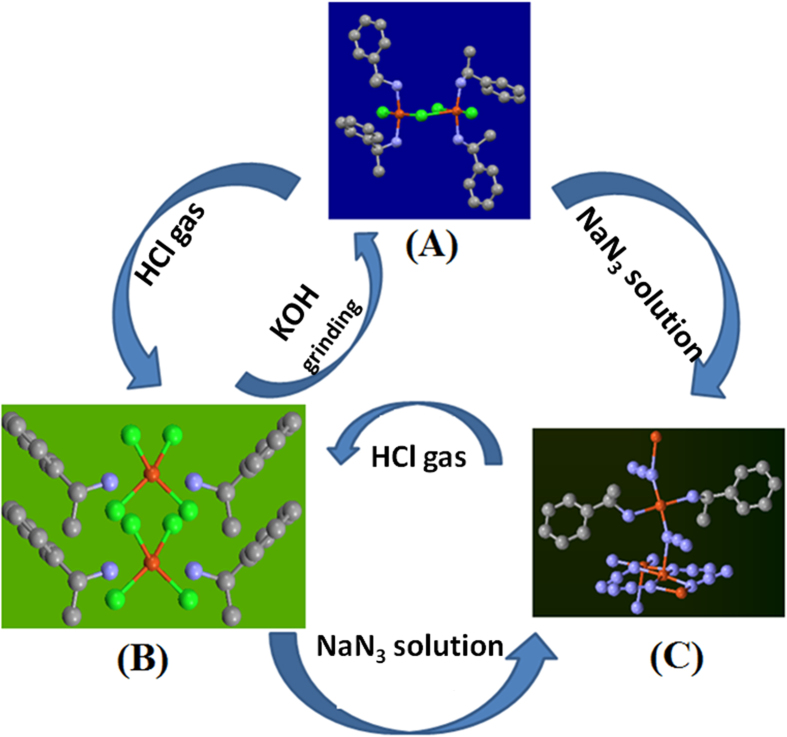
Molecular Accommodative Chemistry with visual colour change: Non-porous Cu(II) complex, (C_6_H_5_CH(CH_3_)NH_2_)_2_CuCl_2_ (**A**) when exposed to HCl gas (solid-gas reaction) reversibly changes to (C_6_H_5_CH(CH_3_)NH_3_)_2_CuCl_4_, double salt (**B**). (**A**) At solid-liquid interface shows Single-Crystal to Single Crystal transformation by selectively absorbing azide anion to form [Cu_3_(C_6_H_5_CH(CH_3_)NH_3_)_2_(N_3_)_6_]_n_ (**C**). (**B**) transforms reversibly to (**C**).

**Figure 2:**
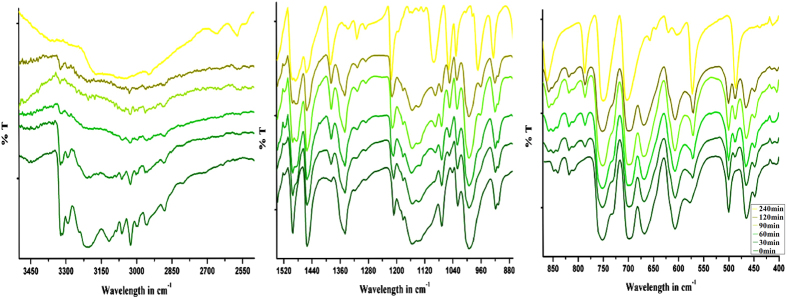
FT-IR study: Monitoring of HCl gas adsorption with time during transformation of A_1_ to B_1_. (colour of the spectra are matching with the colour of the sample during this transformation).

**Figure 3:**
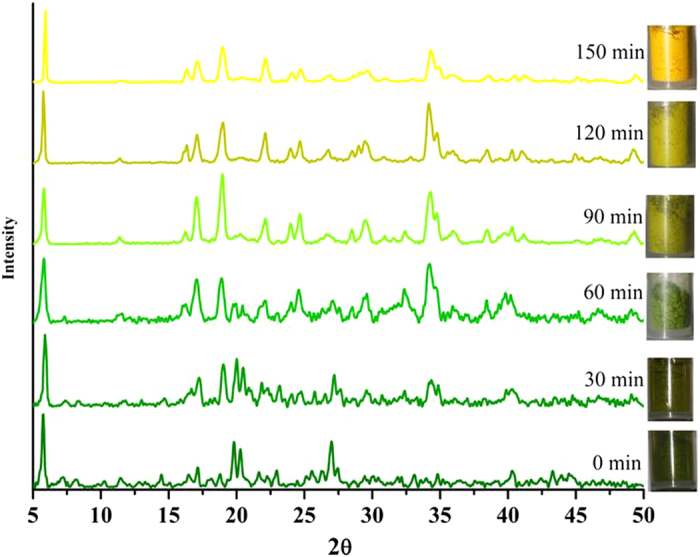
Powder X-ray study: Monitoring of HCl gas adsorption with time during transformation of A_1_ to B_1_. (colour of the spectra are matching with the colour of the sample during this transformation).

**Figure 4:**
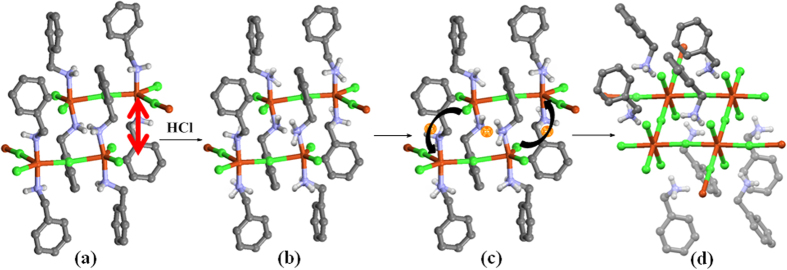
Probable mechanism for transformation of (**a**) 1-D chain of A_1_ to (**d**) 2-D layer of B_1_ through (**b**) benzylammonium formation, and (**c**) charge neutrality driven layer formation at the expense of inversion center between the layers.

**A**_***R***_/**A**_***S***_ absorb HCl gas in crystal to crystal transformation to **B**_***R***_**/B**_***S***_, as shown in Fig. 1. After chemisorptions a unit cell volume of **B**_***R***_**/B**_***S***_ was increased by 16% along with reduction in the crystal packing index (*Z*) from 1 to 2. This process of transformation is monitored using UV-vis. (see Supplementary Fig. S7 online), FT-IR (see Supplementary Fig. S8 online), Powder XRD (see Supplementary Fig. S9–S10 online), CD spectra (see Supplementary Fig. S12 online), and Specific Optical Resolution activity (see Supplementary Table S6-S7 online).

**Figure 5:**
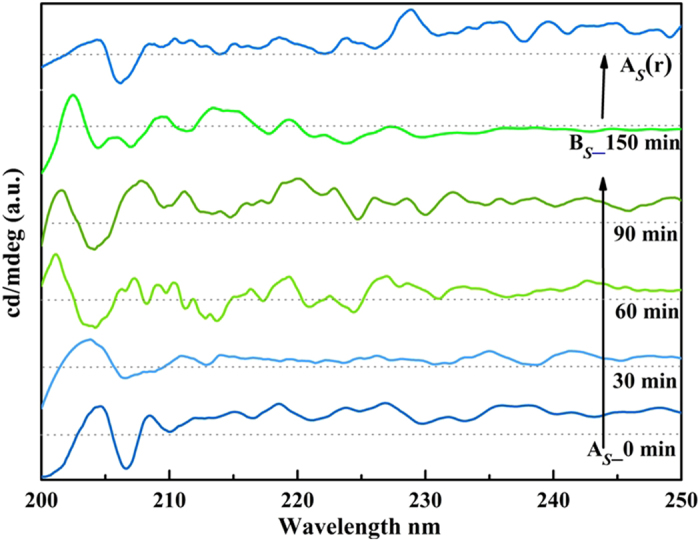
Circular Dichroism study: Monitoring of HCl gas adsorption with time during transformation of A_1_ to B_1_. (colour of the spectra are matching with the colour of the sample during this transformation).

Figure 5 shows no change in cotton effect during CD spectroscopic study during adsorption and desorption by grinding with KOH. This result complements no change in optical rotation, SOR activity, confirming enantiomeric nature of these reversible structural changes (see Supplementary Table S6-S7 online).

Thus, one can say that substitution occur associatively (organic chemists: concerted, similar to S_N_^2^) the crystal. This may be the reason to the observed sustainability of crystalline character during this transformation. This also explain occurrence of spin density only on Cu and Cl atoms, and not on amine N atom, during preliminary DFT instigation on the complex and double salt (see Supplementary Fig. S13 online).

In short, one can design molecular compounds showing adsorption-desorption behavior by simply observing the thermal degradation pathway, as shown for set of compounds in present manuscript, as (see Supplementary Fig. S14 online). In literature, most of the molecules have been studied using TGA, but no emphasis was given for it from the concept of designing these type of compounds.

### Crystal to Crystal transformation at Solid-Gas interface: Ideal Sensor

After adsorption, we observed very clear change in optical property. Recently, we^19^ and other^20^ have shown B structures, A_2_CuCl_4_, exhibit not only interesting molecular magnetic properties but are also multiferroic in nature. While, present set of compounds are no exception for the same, and show distinct changes with interesting magnetic (see Supplementary Fig. S15 online), thermochromic (see Supplementary Fig. S16-S17 online) as well as ferroelectric behavior (see Supplementary Fig. S18 online). Thus, these studies also facilitate novel candidates for multiferroic^21^ and/or multifunctional behavior^22^. We are working on this aspect presently.

### Reversible Crystal to Crystal transformation at Solid-Liquid interfaces: (Molecular Nature due to Self assembly, Molecular Recognition and Anchimeric assistance)

These non-porous molecular compounds, A_*R*_/A_*S*_, selectively accommodate supramolecularly arranged azide anion to form C_*R*_/C_*S*_^23^ (see Supplementary Scheme S3 online), with visible colour change from blue to dark green, as shown in Fig. 1. This transformation is totally different than that is observed at solid-gas interface because azide anion is incorporated with the help of self-assembly of auxiliary ligand. Attached movie (ESI: Movie) further shows this transformation happens with the retention of crystalline character. To the best of our knowledge, this is a first example, where true ‘molecular nature’ is reflected owing to cyclic reversible nature of C_*R*_ to B_*R*_ and A_*R*_ to B_*R*_, as shown in Fig. 1.

C_*R*_/C_*S*_ crystallized in *P*2_1_2_1_2_1_ space group^24^. Here Cu (II) ions are in three crystallographically different environments, two square pyramidal in which each Cu(II) is coordinated with five end-on (*EO*) azide bridges, and one square planar coordinated with nitrogens of two amine ligands and two end-to-end (*EE*) azide bridges (see Supplementary Fig. S19a-S19b online). Self-assembly results into neutral 2D brick-wall layers with a repeating azido-bridged eight membered copper brick (see Supplementary Fig. S19c-S19d online). These C_*R*_ and C_*S*_ compounds have been reported and shown multifunctional properties which include ferromagnetic and weak ferroelectric behaviour. That means azide accommodation can be easily detected by various techniques at solid-liquid interface.

This transformation is driven by ‘self-assembly’ of an auxiliary ligand because chemisorption of azide anion happens with loss of organic ligand in secondary sphere, as shown below:
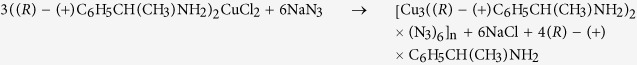
But, in-spite of this ligand loss, true molecular nature of this reaction is remained intact due to cyclic reversible transformations from **C** to **B** and **B** to **A** as shown in Fig. 1 (see Supplementary Scheme S4-S5 online). This means, **C**_***R***_/**C**_***S***_ when exposed to dry HCl vapours forms double salt **B**_***R***_/**B**_***S***_, which in turn can desorbs HCl to give back **A**_***R***_/**A**_***S***_, thus confirming molecularity of the overall reaction (see Supplementary Fig. S21 online). Molecular recognition of this transformation is reflected due to its selective sorption of azide anion, (this is observed mainly for A to C transformation and not for B to C transformation) in presence of other anions, such as Cl^−^, Br^−^, NO_3_^−^, and SCN^−^. Interestingly, solid state CD spectra on the single crystals revealed that this transformation happens with retention of chirality but distinct changes in cotton effect in sensing (see Supplementary Fig. S22-S23 online).

The reason for the observed crystal to crystal transformation (see Supplementary: movie) resides in two factors (a) lattice energy of NaCl crystal might drive reaction 1; (b) concerted-self-assembly can help in directing movement of auxiliary ligand. We associate this crystal to crystal or fast crystallization phenomenon to anchimeric assistance. In organic chemistry, anchimeric assistance is observed when neighbouring atoms/molecule facilitates the activity at the reaction centre.

Normally, the rates of anchimeric assisted reactions are very fast, and happen without racemisation^25^. To support this aspect, experiments with auxiliary ligand in **A**’ (*p*-chloro methyl benzylamine) and **A** (methyl benzylamine) are discussed below.

**Figure 6:**
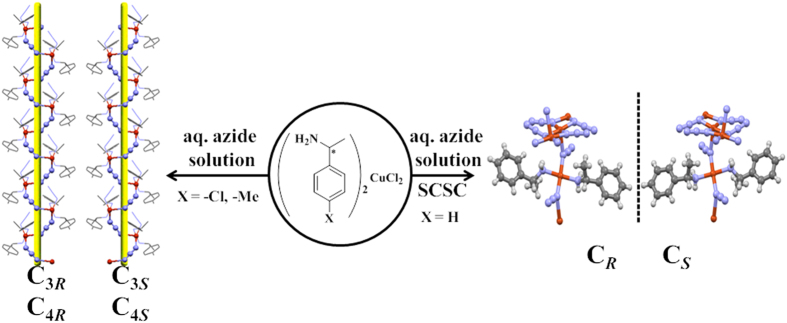
Role of molecules during self-assembly and molecular recognition, true anchimeric assistance during crystal to crystal transformation from A to C.

Figure 6 shows crystal to crystal transformation of A’(A’ exists as polycrystalline) to C’ having 1-dimentional helical chain structure and A to C having 2-dimensional structure during this crystal to crystal transformation^26^. This means auxiliary ligands self-association help in molecular recognition of azide anion and hence crystallization in different dimensional structure. The fast rate of crystallization observed during present crystal to crystal transformation can be correlated only to anchimeric assistance. Therefore, anchimeric assistance, a new molecular force along with molecular recognition and self-assembly process will definitely help in understanding rate of crystallization or crystal to crystal transformation in molecular compounds, and complexes.

## Conclusion

This report coveys that non-porous material can act as a sensor material at both solid-gas and solid-liquid interface. The concept focused on ‘accommodative’ behavior of central atom/ion and ‘breathing’ of ligand field will certainly help in revealing basic coordination chemistry and in particular crystal to crystal transformations. In short, present work will generate new dimension in designing smart materials.

## Methods

### Materials

All chemicals and solvents were of analytical grade reagents. Unless stated otherwise, all reagents were purchased from Aldrich Chemicals and used without further purification. The following chemicals were used in the present study. Benzylamine, 4-chloro benzylamine, (*R*)-(+)-*α*-ethyl phenyl amine; (*S*)-(−)-*α*-ethyl phenyl amine, (*R*)-(+)-α-Ethyl benzylamine; (*S*)-(−)-α-Ethyl benzylamine; (*R*)-(+)-4-Chloro-*α*-methylbenzylamine; (*S*)-4-Chloro-*α*-methylbenzylamine; (*R*)-(+)-4-methyl-*α*–methylbenzylamine; (*S*)-N-Methyl-1-phenylethanamine; CuCl_2_.2H_2_O; Conc. HCl: Loba Chemie; Conc. H_2_SO_4_: Merck; Sodium Azide: Qualigens; Ethanol: Baroda Chemicals; Deionized water.

#### Solid gas interface

Sample vial containing crystals were kept in dry HCl gas chamber. The reaction took place over a time with observable colour change.

#### Solid liquid interface

Crystals kept on a filter paper, which is dipped in a watch glass containing saturated aqueous solution of sodium azide (1 M) for 5 seconds. Detailed procedure is available in Supplementary.

### Characterisation

Elemental analyses were determined using a Perkin Elmer Series II 2400 elemental analyzer. The IR spectra were recorded in the 4000–400 cm-1 region using KBr pellets and a Perkin Elmer RX1 Spectrophotometer. The solid-state UV spectra were recorded on a Perkin Elmer Lambda 35 Spectrophotometer with KBr pellets. The solid-state circular dichroism (CD) spectra were recorded on a Jasco J-851-150 L CD spectropolarimeter in Nujol. Electron Spin Resonance measured on crystalline samples was performed on an ESR-Varian (E-112) Spectrometer. Thermogravimetric analyses (TG-DTA) were performed single crystals samples using SII TG/DTA 6300 EXSTAR Analyser under N_2_ atmosphere with a heating rate of 10 °C/min. For reaction monitoring (A_1_ to B_1_ and A_*S*_ to B_*S*_), Powder X-ray diffraction were collected in the 2*θ* range 5–50° at 300 K for polycrystalline samples on Philips X’pert MPD System. All of the bulk polycrystalline samples were ground in an agate mortar and pestle and filled into 0.5 mm glass capillaries and recorded on ‘Xcalibur, Eos, Gemini’ Diffractometer in the 2*θ* range 5–50° at 300 K. Specific optical rotation (SOR) activity was recorded on Jasco P-2000 Polarimeter with sodium source.

### X-ray Crystallography

Single-crystal data of **B**_***R***_ and **B**_***S***_ were collected on a Bruker Smart 1000 CCD Diffractometer, with Mo KR radiation (ì) 0.710 73 Å). All empirical absorption corrections were applied by using the SADABS program-14. The structures were solved using direct methods, which yielded the positions of all non-H atoms. These were refined first isotropically and then anisotropically. All of the H atoms of the ligands were placed in calculated positions with fixed isotropic thermal parameters and included in the structure factor calculations in the final stage of full-matrix least-squares refinement. All calculations were performed using the SHELXTL system of computer programs.15.

## Additional Information

**How to cite this article**: Mande, H. M. and Ghalsasi, P. S. Accommodative Behavior of Non-porous Molecular crystal at Solid-Gas and Solid-Liquid Interface. *Sci. Rep.* **5**, 14460; doi: 10.1038/srep14460 (2015).

## Supplementary Material

Supplementary Information

## References

[b1] Abellán G. *et al.* Stimuli-responsive hybrid materials: breathing in magnetic layered double hydroxides induced by a thermoresponsive molecule. Chem. Sci. 6, 1949 (2015). 10.1039/c4sc03460k PMC5495995 28706645

[b2] Garcia-Amorós J. & Velasco D. in Responsive Materials and Methods 27–58 (John Wiley & Sons, Inc. 2013).

[b3] Kreno L. E. *et al.* Metal-Organic Framework Materials as Chemical Sensors. Chem. Rev. 112, 1105–1125 (2012). 22070233 10.1021/cr200324t

[b4] Czaja A. U., Trukhan N. & Muller U. Industrial applications of metal-organic frameworks. Chem. Soc. Rev. 38, 1284–1293 (2009). 19384438 10.1039/b804680h

[b5] Kuppler R. J. *et al.* Potential applications of metal-organic frameworks. Coord. Chem. Rev. 253, 3042–3066 (2009).

[b6] Farrusseng D., Aguado S. & Pinel C. Metal–Organic Frameworks: Opportunities for Catalysis. Angew. Chem. Int. Ed. 48, 7502–7513 (2009). 10.1002/anie.200806063 19691074

[b7] Gale P. A. & Caltagirone C. Anion sensing by small molecules and molecular ensembles. Chem. Soc. Rev. 44, 4212–4227 (2015). 24975326 10.1039/c4cs00179f

[b8] Coronado E., Giménez-Marqués M., Gómez-García C. J. & Espallargas G. M. Dynamic Magnetic Materials Based on the Cationic Coordination Polymer [Cu(btix)_2_]_n_^2n+^ [btix = 1,4-Bis(triazol-1-ylmethyl)benzene]: Tuning the Structural and Magnetic Properties through Anion Exchange. Inorg. Chem. 51, 12938–12947 (2012). 23148577 10.1021/ic302066e

[b9] Chen B., Wang L., Zapata F., Qian G. & Lobkovsky E. B. A Luminescent Microporous Metal–Organic Framework for the Recognition and Sensing of Anions. J. Am. Chem. Soc. 130, 6718–6719 (2008). 18452294 10.1021/ja802035e

[b10] Beer P. D. & Gale P. A. Anion recognition and sensing: the state of the art and future perspectives. Angew. Chem. Int. Ed. 40, 486–516 (2001). 11180358

[b11] Kitagawa S., Kitaura R. & Noro Shin-ichiro. Functional Porous Coordination Polymers. Angew. Chem. Int. Ed. 43, 2334–2375 (2004). 10.1002/anie.200300610 15114565

[b12] Adams C. J., Colquhoun H. M., Crawford P. C., Lusi M. & Orpen A. G. Solid-State Interconversions of Coordination Networks and Hydrogen-Bonded Salts. Angew. Chem. Int. Ed. 46, 1124–1128 (2007). 10.1002/anie.200603593 17191295

[b13] Coronado E., Giménez-Marqués M., Espallargas G. M. & Brammer L. Tuning the magneto-structural properties of non-porous coordination polymers by HCl chemisorption. Nat. Commun. 3, 828 (2012). 22569372 10.1038/ncomms1827

[b14] Vishwakarma A. K. & Ghalsasi P. S. Thermal decomposition paths in A_2_CuCl_4_ complexes: anilinium and its derivatives. J. Therm. Anal. Calorim. 107, 155–158 (2012).

[b15] Zi Guofu, Xiang Li, Zhang Yadong, Wang Qiuwen & Zhang Zhanbin. Synthesis, structure, and activity of (PhCH_2_NH_2_)_2_CuCl_2_for oxidative coupling of 2-naphthylamine. Appl. Organometal. Chem. 21, 177–182 (2007).

[b16] Vishwakarma A. K. *Studies on multifunctional organic-inorganic hybrid compounds* Ph.D. Thesis, The M.S. University of Baroda, India (2013).

[b17] Mínguez Espallargas G. *et al.* Reversible gas uptake by a nonporous crystalline solid involving multiple changes in covalent bonding. J. Am. Chem. Soc. 129, 15606–15614 (2007). 18034480 10.1021/ja075265t

[b18] Luo Mei Sun, Jie Zhou, Shi-Ming Yin Hao & Ke-Liang Hu. The crystal structures of chiral (*R/S*)(C_8_H_11_N)_2_CuCl_2_. Res Chem Intermed 36, 1049–1054 (2010).

[b19] Vishwakarma A. K., Ghalsasi P. S., Navamoney A., Lan Y. & Powell A. Structural phase transition and magnetic properties of layered organic–inorganic hybrid compounds: p-Haloanilinium tetrachlorocuparate (II). Polyhedron 30, 1565–1570 (2011).

[b20] Polyakov A. O. *et al.* Coexisting ferromagnetic and ferroelectric order in a CuCl4-based organic–inorganic hybrid. Chem. Mater. 24, 133–139 (2011).

[b21] Caretta A. *et al.* Low-frequency Raman study of the ferroelectric phase transition in a layered CuCl 4-based organic-inorganic hybrid. Phys. Rev. B 89, 024301 (2014).

[b22] Pardo E. *et al.* Multiferroics by Rational Design: Implementing Ferroelectricity in Molecule‐Based Magnets. Angew. Chem. 124, 8481–8485 (2012). 10.1002/anie.201202848 22767449

[b23] Supriya S. & Das S. K. Solid-to-solid formation at the solid–liquid interface leading to a chiral coordination polymer from an achiral monomer. Chem. Commun. 47, 2062–2064 (2011). 10.1039/c0cc04375c 21206950

[b24] Gu Z.-G., Song Y., Zuo J.-L. & You X.-Z. Chiral Molecular Ferromagnets Based on Copper(II) Polymers with End-On Azido Bridges. Inorg. Chem. 46, 9522–9524 (2007). 17939656 10.1021/ic7015325

[b25] March J. Advanced organic chemistry: reactions, mechanisms, and structure. Vol. 4 (McGraw-Hill: New York, , 1968).

[b26] Mande H. M., Ghalsasi P. S. & Arulsamy N. Interplay of chiral auxiliary ligand and azide bridging ligand during the coordination network formation with Copper (II). Cryst. Growth Des. 14, 4254–4257 (2014).

