# Supplementary material for: Accommodative Behavior of Non-porous Molecular crystal at Solid-Gas and Solid-Liquid Interface
Source: Sci Rep. 2015 Sep 28;5:14460. doi: 10.1038/srep14460 (PMC4585990; doi:10.1038/srep14460)
Supplement: Supplementary Information [file srep14460-s1.pdf]

Supporting Information

Accommodative ‘Molecular’ Behavior of Non-porous Molecular crystal at Gas and Liquid Interface

Hemant M. Mande [a] **and Prasanna S. Ghalsasi*** [a]

[a] Department of Chemistry, Faculty of Science, The Maharaja Sayajirao University of Baroda ,Vadodara, India.; Tel:+919427849728 ; E-mail: prasanna.ghalsasi@gmail.com

*To whom correspondence should be addressed. E-mail: prasanna.ghalsasi@gmail.com

Table of contents

Page No.

1	**General Experimental Section**	S5
2	**Synthesis and Experimental Section**	S6
2.1	Synthesis of A_1, A_R,A_S (Scheme S1)	S6
2.2	Transformation of B_1/B_R/B_S from A_1/A_R/A_S (Scheme S2)	S8
2.3	Transformation of C_R/C_S from A_R/A_S (Scheme S3)	S9
2.4	Transformation of C_R/C_S from B_R/B_S (Scheme S4)	S10
2.5	Transformation of B_R/B_S from C_R/C_S (Scheme S5)	S11
3	**Crystal to Crystal transformation Solid-Gas interface**	S12
3.1	X-ray crystal structure of A_1 and B_1 (Figure S1)	S12
3.2	X-ray crystallography data of B_1(Table S1)	S13
3.3	Inner coordination and outer coordination sphere (Table S2)	S14
3.4	Comparison of observed XRPD pattern of bulk sample and simulated XRPD pattern (from single crystal data)	S15
3.4.1	Observed and Simulated Powder X-Ray Diffraction Patterns of B_1 (Figure S2)	S15
3.4.2	Observed (KOH treatment-A_{1r}) and Simulated Powder X-Ray Diffraction Patterns of A_1(Figure S3)	S15
3.5	Planes {h k l} passing through different 2θ values in A_1 (Table S3)	S16
4	**Accommodative nature at Solid-Gas interface: Stereochemistry of Crystal to Crystal transformation**	S17
4.1	$A_S \rightarrow B_S$ transformation (Figure S4)	S17
4.1	X-ray crystal structure of A_R/ A_S (Figure S5)	S17
4.2	X-ray crystal structure of B_R/ B_S (Figure S6)	S18
4.3	X-Ray crystallography data for B_R and B_S (Table S4)	S19

4.4 Inner coordination and outer coordination sphere (Table S5) S20

4.5 Solid state absorption spectra of conversion of $A_S \rightarrow B_S$ (Figure S7) S21

4.6 *ex-situ* FT-IR monitoring of $A_R/A_S \rightarrow B_R/B_S$(Figure S8) S22

4.7 Powder XRD S24

4.7.1 Simulated XRPD pattern of B_S (blue), observed data of bulk sample (black) (Figure S9) S24

4.7.2 *ex-situ* reaction monitoring with time of conversion of A_S to B_S(Figure S10) S24

4.7.3 Observed (KOH treatment- A_S r) and Simulated Powder X-Ray Diffraction Patterns of A_S (Figure S11) S25

4.8 Solid CD spectra of A_R, A_S, B_R and B_S in nujol (Figure S12) S26

4.9 Specific Optical Rotation S27

4.9.1 SOR data of s A_R and A_S, B_R and B_S in methanol at 30ºC (Table S6) S27

4.9.2 *ex-situ* reaction monitoring with time of conversion of A_S to B_S using SOR in methanol and S1(r) showed SOR of conversion of $B_S \rightarrow A_S$ at 30ºC (Table S7) S27

4.10 Theoretical study on A_S and B_S(Figure S13) S29

4.11 Different structures of ligands (Figure S14) S30

4.12 Solid state EPR spectra of A_S and B_S at room temperature (Figure S15) S31

4.13 DSC & Thermochromic behavior for B_1(Figure S16) S32

4.14 DSC & Thermochromic behavior for B_R / B_S(Figure S17) S33

4.15 PE Loop measurement of B_R (Figure S18) S34

5 Crystal to Crystal transformation at Solid-Liquid interface: Self assembly, Molecular Recognition and Anchimeric assistance S35

5.1 X-ray crystal structure of C_R, C_S and its crystal packing (Figure S19) S35

5.2 Powder XRD S36

5.2.1 Comparison of observed XRPD pattern of bulk sample and simulated XRPD pattern (from single crystal data)of C_R/C_S(Figure S20) S36

5.2.2 Comparison of observed XRPD pattern of bulk sample and simulated XRPD pattern (from single crystal data) of C_R/C_S and B_R/B_S (Figure S21) S36

5.3 Solid state CD spectra S37

5.3.1 Solid state CD spectra of A_R, A_S, C_R and C_S (Figure S22) S37

5.3.2 Solid state CD spectra of B_R, B_S, C_R and C_S in nujol (Figure S23) S37

6 Indexed Powder XRD S38

PXRD of A_1 and B_1 S38

PXRD of $A_{1(r)}$ and A_R S39

PXRD of B_R and $A_{R(r)}$ S40

1. General Experimental Section

All chemicals and solvents were of analytical grade reagents. Unless stated otherwise, all reagents were purchased from Aldrich Chemicals and used without further purification.

Elemental analyses were determined using a Perkin Elmer Series II 2400 elemental analyzer. The IR spectra were recorded in the 4000-400 cm-1 region using KBr pellets and a Perkin Elmer RX1 Spectrophotometer. The solid-state UV spectra were recorded on a Perkin Elmer Lambda 35 Spectrophotometer with KBr pellets. The solid-state circular dichroism (CD) spectra were recorded on a Jasco J-851-150 L CD spectropolarimeter in Nujol. Electron Spin Resonance measured on crystalline samples was performed on an ESR-Varian (E-112) Spectrometer. Thermogravimetric analyses (TG-DTA) were performed single crystals samples using SII TG/DTA 6300 EXSTAR Analyser under N_2 atmosphere with a heating rate of 10ºC/min. For reaction monitoring (A_1 to B_1 and A_S to B_S), Powder X-ray diffraction were collected in the 2θ range 5-50º at 300K for polycrystalline samples on Philips X'pert MPD System. All of the bulk polycrystalline samples were ground in an agate mortar and pestle and filled into 0.5 mm glass capillaries and recorded on 'Xcalibur, Eos, Gemini' Diffractometer in the 2θ range 5-50º at 300K. Specific optical rotation (SOR) activity was recorded on Jasco P-2000 Polarimeter with sodium source.

X-ray Crystallography

Single-crystal data of $\mathbf{B}_{\boldsymbol{R}}$ and $\mathbf{B}_{\boldsymbol{S}}$ were collected on a Bruker Smart 1000 CCD Diffractometer, with Mo KR radiation (ì) 0.710 73 Å). All empirical absorption corrections were applied by using the SADABS program-14. The structures were solved using direct methods, which yielded the positions of all non-H atoms. These were refined first isotropically and then anisotropically. All of the H atoms of the ligands were placed in calculated positions with fixed isotropic thermal parameters and included in the structure factor calculations in the final stage of full-matrix least-squares refinement. All calculations were performed using the SHELXTL system of computer programs.15.

2. Synthesis and Experimental Section

2.1 Synthesis of $A_1/A_R/A_S$

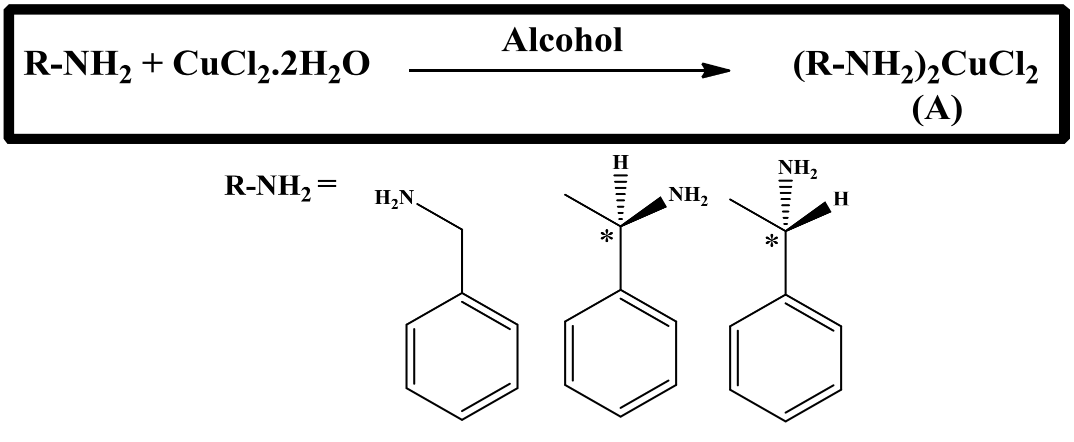

A_1 A_R A_S

Scheme S1:

To a 3.0 mL (23.2 mmol) of benzylamine (**a_1**) in a dry 100 mL flask, 1.7048 g (10.0 mmol) $CuCl_2.2H_2O$ was added under moisture and air-free conditions. The mixture was dissolved in 30 mL of dry ethanol and refluxed for 24 h. The solvent was reduced under vacuum causing precipitation to green solid. Slow evaporation from saturated ethanolic solution afforded 0.88 g of green crystals of **A_1**. Yield:- 53.4%,

A_R and **A_s** were obtained using a similar method to that of **A_1**, except that ligands (*R*)-(+)- ethyl phenyl amine (**a_{1R}**) and (*S*)-(-)-α-methyl benzylamine (**a_{1S}**) were used. Yield: 45-55%.

A_1:Anal. Calc. for $C_{14}H_{18}Cl_2CuN_2$: C, 48.21; H, 5.20; N, 8.03%. **Found:** C, 48.11; H, 5.29; N, 7.83%; **FT-IR (KBr):** 3322 (vs), 3313 (vs), 3292 (s), 3246 (m), 3210 (s), 3117 (s), 3062 (m), 3025 (s), 2997 (m), 2957 (m), 2880 (m), 2800 (w), 2068 (m), 1966 (m), 1947 (s), 1878 (m), 1804 (m), 1666 (m), 1601 (s), 1575 (vs), 1555 (s), 1494 (ssh), 1346 (vs), 1317 (w), 1285 (w), 1207 (m), 1155 (vs), 1134 (vs), 1097 (s), 1071 (m), 1024 (m), 995 (vssh), 917 (s), 908 (s), 842 (m), 819 (m), 752 (vssh), 729 (s), 697 (vs), 668 (vs), 608 (vs), 578 (s), 500 (s), 463 (vs), 448 (s) cm^{-1}.

A_R: Anal. Calc. for $C_{16}H_{22}N_2CuCl_2$: C, 50.73; H, 6.39; N, 7.39%. **Found:** C, 50.79; H, 6.26; N, 7.43%; **FT-IR (KBr):** 3321 (m), 3301 (s), 3250 (w), 3220 (s), 3025 (vs), 2966 (vs), 2916 (s), 2722 (w), 2772 (w), 2765 (m), 2480 (s), 2032 (m), 1971 (w), 1951 (w), , 1887 (w), 1810 (w), 1589 (vs), 1568 (s), 1516 (s), 1496 (ssh), 1456 (ssh), 1383 (vs), 1336 (w), 1291 (m), 1226 (s), 1193 (s), 1155 (vs), 1126 (s), 1090 (vs), 1071 (s), 1028 (m), 1007 (w), 986 (m), 917 (m), 891

(w), 875 (w), 772 (s), 764 (s), 749 (s), 699 (vssh), 656 (w), 604 (m), 580 (w) and 528 (m) cm^{-1}. **A*R***: $[\alpha]_D^{25}$ = +81.78 (c 0.0428, THF) for ((*R*)-(+)-α-ethyl phenyl amine)$_2$CuCl$_2$.

A*S*: Anal. Calc. for $C_{16}H_{22}N_2CuCl_2$: C, 50.73; H, 6.39; N, 7.39%. **Found:** C, 50.76; H, 6.31; N, 7.34%; **FT-IR (KBr):** 3322 (s), 3303 (m), 3250 (w), 3222 (s), 3140 (w), 3026 (vs), 2968 (vs), 2915 (s), 2720 (w), 2772 (w), 2767 (m), 2481 (s), 2031 (m), 1971 (w), 1952 (w), , 1885 (w), 1810 (w), 1591 (vs), 1567 (s), 1516 (s), 1495 (ssh), 1456 (ssh), 1384 (vs), 1336 (w), 1315 (w), 1291 (m), 1228 (s), 1194 (s), 1153 (vs), 1126 (s), 1089 (vs), 1071 (s), 1051 (w), 1029 (m), 1005 (w), 987 (m), 915 (m), 891 (w), 879 (w), 770 (s), 761 (s), 749 (s), 698 (vssh), 656 (w), 617 (w), 604 (m), 583 (w) and 527 (m) cm^{-1}. **A*S***: $[\alpha]_D^{25}$ = -81.71 (c 0.0428, THF) for ((*S*)-(-)-α-ethyl phenyl amine)$_2$CuCl$_2$.

2.2 Transformation of B_1/B_R/B_S from A_1/A_R/A_S

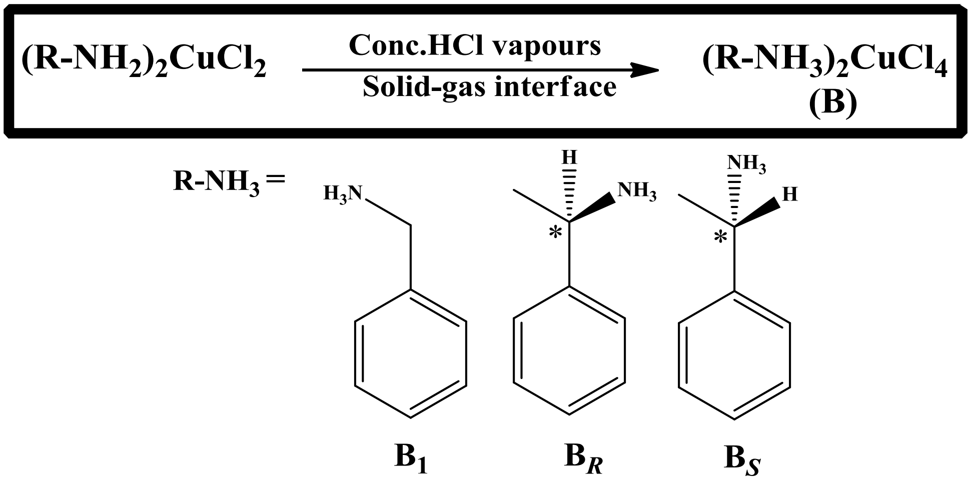

Scheme S2:

The dry HCl gas was passed in a sample vial containing **A_1/A_R/A_S** (1 g). The reaction takes place in 2-3 hrs with observable color change to obtain **B_1/B_R/B_S** respectively.

Yield:- 100%

B_1: Anal. Calc. for $C_{14}H_{20}Cl_4CuN_2$: C, 39.88; H, 4.78; N, 6.61%. **Found:** C, 39.92; H, 4.70; N, 6.71%; **FT-IR (KBr):** 3154 (vs), 2885 (s), 2574 (m), 2350 (w), 2301 (w), 1958 (w), 1774 (m), 1566 (s), 1488 (s), 1456 (m), 1389 (m), 1313 (w), 1217 (s), 1098 (s), 1050 (m), 1031 (w), 968 (w), 924 (w), 864 (w), 787 (w), 754 (ssh), 701 (ssh), 669 (w), 573 (m) and 486 (m) cm^{-1}.

B_R: Anal. Calc. for $C_{16}H_{24}N_2CuCl_4$: C, 42.73; H, 5.38; N, 6.23%. **Found:** C, 42.69; H, 5.28; N, 6.18%; **FT-IR (KBr):** 2946 (vs), 2656 (m), 1593 (vs), 1564 (s), 1494 (vs), 1454 (vs), 1386 (vs), 1370 (m), 1334 (w) 1314 (m), 1288 (m), 1222 (vs), 1161 (m), 1083 (s), 1058 (s), 1029 (m), 970 (s), 918 (m), 766 (vssh), 749 (m), 697 (vssh), 536 (vssh) and 476 (m) cm^{-1}.

B_S: Anal. Calc. for $C_{16}H_{24}N_2CuCl_4$: C, 42.73; H, 5.38; N, 6.23%. **Found:** C, 42.42; H, 5.44; N, 6.28%; **FT-IR (KBr):** 3054 (vs), 2656 (m), 1593 (vs), 1564 (s), 1493 (vs), 1454 (vs), 1386 (vs), 1370 (m), 1334 (w), 1314 (m), 1288 (m), 1222 (vs), 1161 (m), 1083 (s), 1058 (s), 1029 (m), 970 (s), 918 (m), 766 (vssh), 749 (m), 697 (vssh), 537 (vssh) and 476 (m) cm^{-1}.

2.3 Transformation of C_R/C_S from A_R/A_S (Single crystal to single crystal transformation)

$$(R\text{-}NH_2)_2CuCl_2 + NaN_3 \xrightarrow[\text{Solid-liquid interface}]{\text{Water}} [Cu(R\text{-}NH_2)_2(N_3)_6]_n \; \textbf{(C)}$$

$R\text{-}NH_2$ =

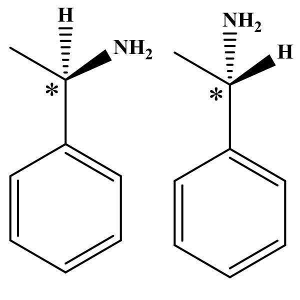

C_R C_S

Scheme S3:

Crystals of $\mathbf{A_R}$/$\mathbf{A_S}$ on filter paper dipped in a watch glass containing saturated aqueous solution of sodium azide (1M) for 5 seconds. The color of the $\mathbf{A_R}$/$\mathbf{A_S}$ changes from blue to green giving crystals of $\mathbf{C_R}$/$\mathbf{C_S}$.

C_R: Anal. Calc. for $C_{16}H_{22}N_{20}Cu_3$: C, 28.05; H, 3.24; N, 40.89%. **Found:** C, 27.80; H, 3.30; N, 40.65%; **FT-IR (KBr):** 3350 (m), 3317 (s), 3298(w), 3237(s), 3210 (s), 3138 (s), 3083(w), 3028 (m), 3006 (w), 2965 (s), 2921 (w), 2867 (w), 2077 (ssh), 2040 (ssh), 1588 (s), 1573 (vs), 1493 (m), 1453 (vs), 1388 (s), 1374 (s), 1341 (s), 1306 (m), 1287 (m), 1240 (s), 1221 (s), 1150 (vssh), 1089 (vssh), 1074 (vs), 1064 (vs), 1043 (s), 1024 (s), 1003 (s), 994 (s), 926 (w), 910 (m), 888 (s), 782 (m), 757 (vs), 708 (s), 698 (ssh), 670 (vs), 603 (s), 561 (m), 543 (m), 529 (w), 474 (w) and 441 (w) cm^{-1}.

C_S: Anal. Calc. for $C_{16}H_{22}N_{20}Cu_3$: C, 28.05; H, 3.24; N, 40.89%. **Found:** C, 27.85; H, 3.38; N, 40.59%; **FT-IR (KBr):**3351 (m), 3315 (s), 3295(w), 3232(s), 3212 (s), 3138 (s), 3058(m), 3029 (m), 2961 (s), 2924 (w), 2075 (ssh), 2042 (ssh), 1586 (s), 1571 (vs), 1491 (m), 1456 (vs), 1387 (s), 1371 (s), 1342 (s), 1308 (m), 1290 (m), 1237 (s), 1218 (s), 1191 (w), 1148 (vssh), 1091 (vssh), 1071 (vs), 1067 (vs), 1042 (s), 1021 (s), 1003 (s), 994 (s), 926 (w), 910 (m), 900 (w), 888 (s), 848 (w), 782 (m), 769 (w), 757 (vs), 710(s), 695 (ssh), 672 (vs), 602 (s), 561 (m), 542 (m), 530 (w), 473 (w) and 442 (w) cm^{-1}.

2.4 Transformation of C_R/C_S from B_R/B_S

$$(R\text{-}NH_3)_2CuCl_4 \xrightarrow[\text{Solid-liquid interface}]{\text{Aq. azide sol.}} [Cu(R\text{-}NH_2)_2(N_3)_6]_n \; \textbf{(C)}$$

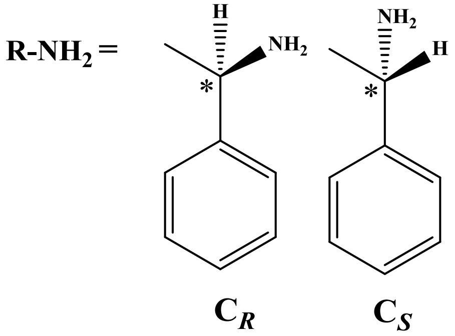

Scheme S4:

Crystals of $\mathbf{B}_R/\mathbf{B}_S$ filter paper dipped in a watch glass containing saturated aqueous solution of sodium azide (1M) for 5 seconds. The color of the $\mathbf{B}_R/\mathbf{B}_S$ changes from yellow green to dark green giving powder sample of $\mathbf{C}_R/\mathbf{C}_S$.

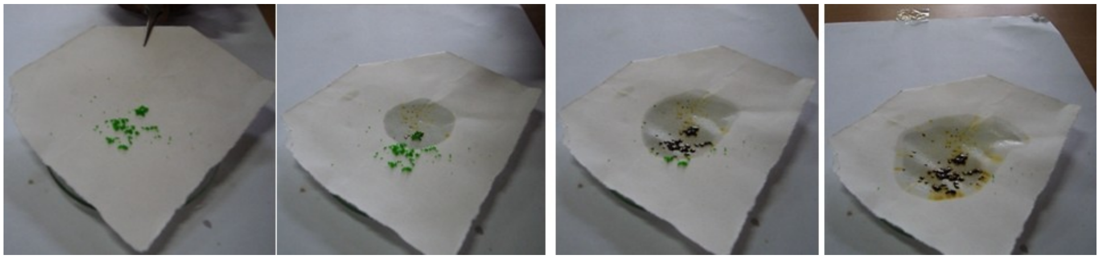

(a) **(b)** **(c)** **(d)**

Crystal transformation of $\mathbf{B}_R/\mathbf{B}_S \rightarrow \mathbf{C}_R/\mathbf{C}_S$

2.4 Transformation of B_R/B_S from C_R/C_S

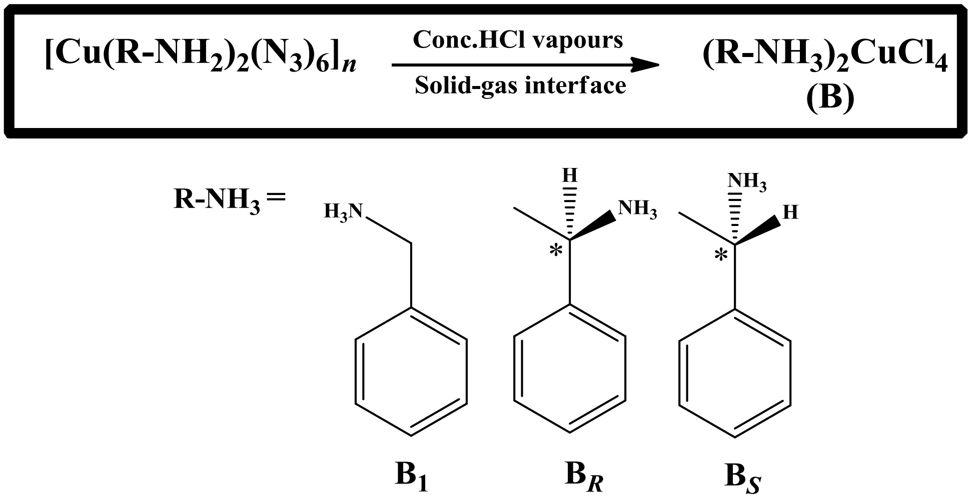

Scheme S5:

Sample vial containing crystals of **C_R/C_S** (1 g) was kept in HCl gas chamber. The reaction took place over a 10-12 hrs to form **B_R/B_S** with observable color change from dark green to yellow green.

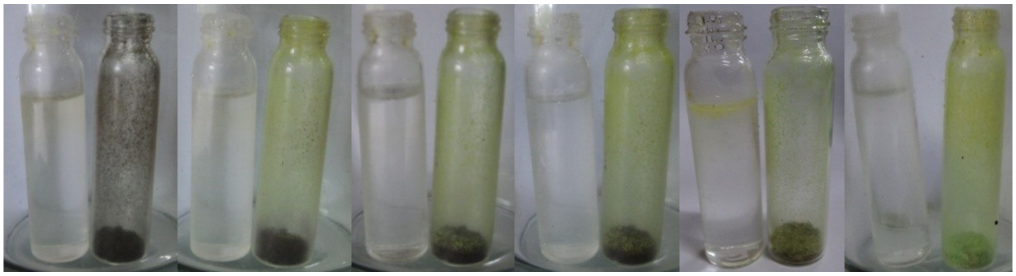

Crystal transformation of $C_R/C_S \rightarrow B_R/B_S$

3. Crystal to Crystal transformation Solid-Gas interface

3.1 X-ray crystal structure of A_1 and B_1

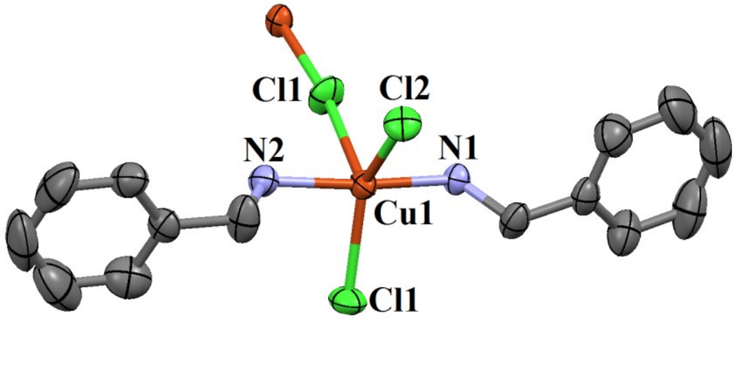

(a)

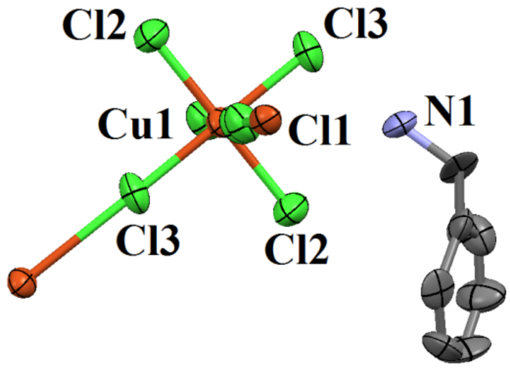

(b)

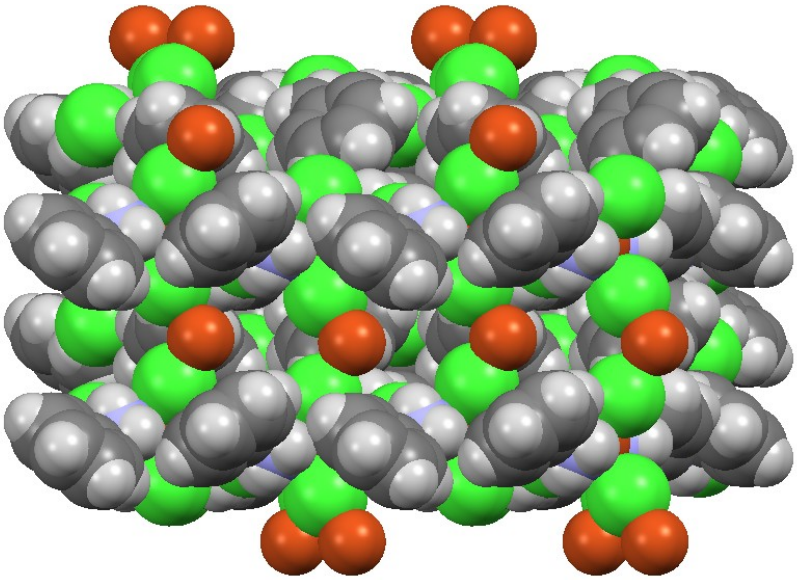

(c)

Figure S1: Molecular view of (a) $\mathbf{A_1}$ and (b) $\mathbf{B_1}$ (thermal ellipsoid are shown at 50 % probability) ; (c) A_1 with space filled style denotes the absence of porosity.

Table S1: X-ray crystal data of B_1

	*B_1
**CCDC**	1015762
Empirical formula	$C_{28}H_{40}Cl_8Cu_2N_4$
Formula weight	843.32
Wavelength (Å)	1.54184
Crystal system	Triclinic
Space group	P1
a (Å)	10.4498(12)
b (Å)	10.4921(12)
c (Å)	15.9114(18)
α/°	81.218(3)
β/°	80.432(3)
γ/°	89.237(3)
V ($Å^3$)	1700.0(3)
Z	2
Dcalc (Mg/m^3)	1.648
Reflections collected	20181
Independent reflections	11016 [R_{int} = 0.0491, R_{sigma} = 0.0839
Goodness-of-fit on F^2	0.908
Data / restraints / Parameters	1707/6/160
Final R indices [I>2α(I)]	R_1 = 0.0771, wR_2 = 0.2121
R indices (all data)	R_1 = 0.1067, wR_2 = 0.2373

*Sabine *et. al*. Chem. Eur. J. 2013, 19, 5342 – 5351.

3.3 Inner coordination and outer coordination sphere

TableS2: Bond lengths of A_1 and B_1

	**A_1 (Å)**	**B_1 (Å)**
**Cu (1)-N(1)**	1.983(3)	
**Cu (1)-N(2)**	1.984(3)	
**Cu (1)-Cl(1)**	2.340(11)	2.289(2)
**Cu (1)-Cl(1#)**	2.629(11)	2.289(2)
**Cu (1)-Cl(2)**		2.293(2)
**Cu (1)-Cl(2#)**		2.298(2)
**Cu (1)-Cl(3)**	2.299(10)	

3.4 Comparison of observed XRPD pattern of bulk sample and simulated XRPD pattern (from single crystal data)

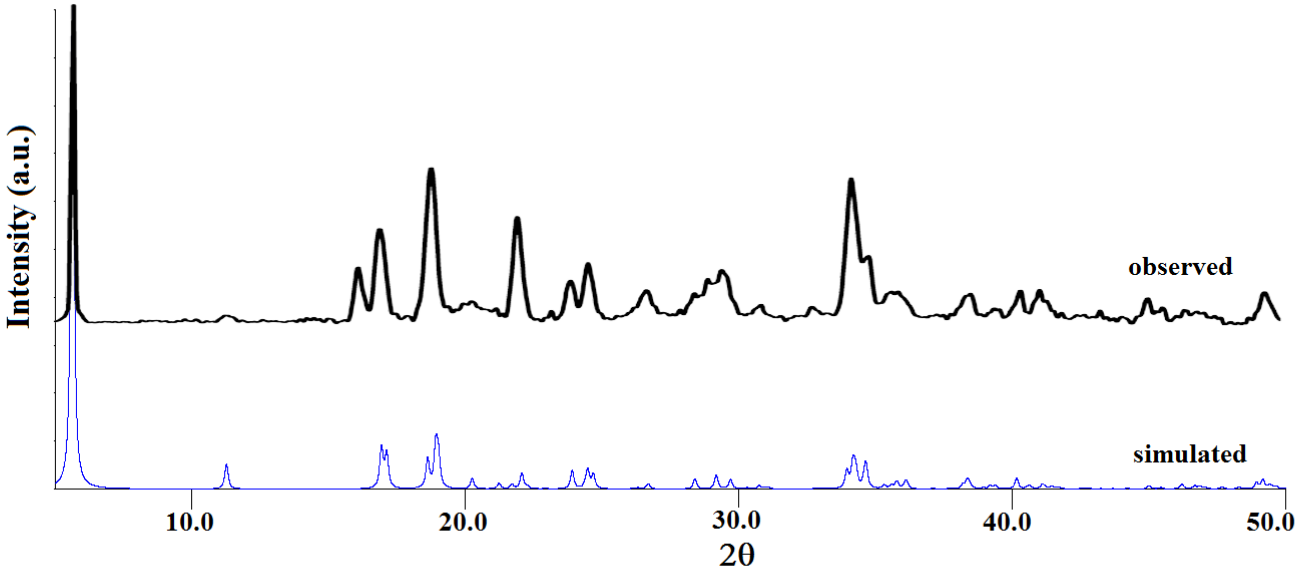

Fig. S2 Observed and Simulated Powder X-Ray Diffraction Patterns of B_1

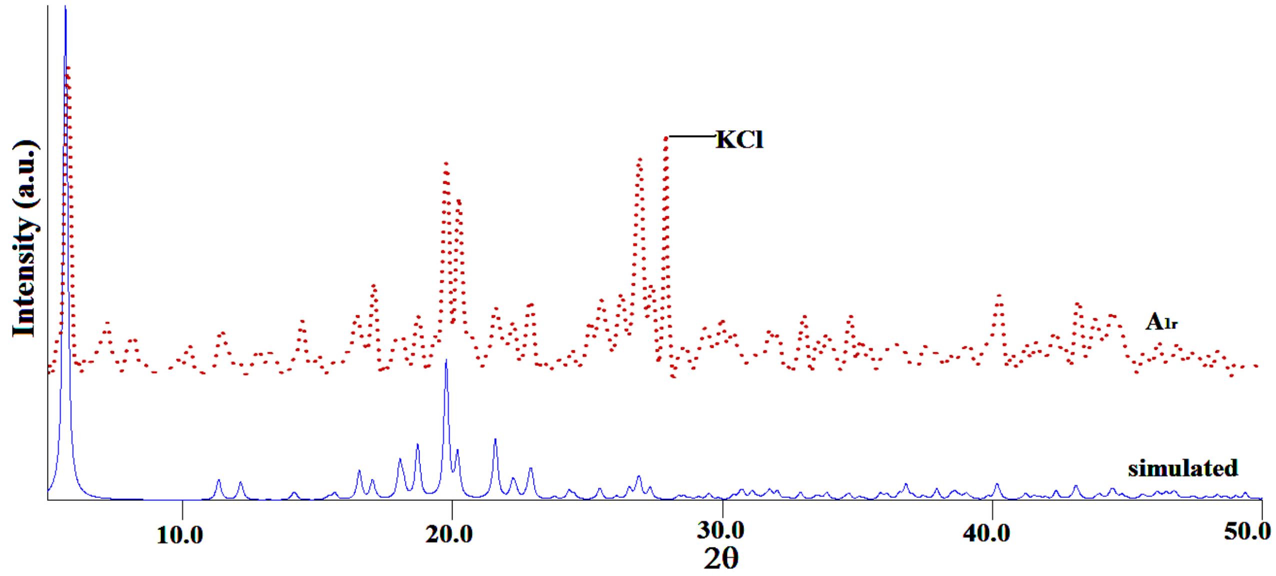

Fig.S3 Observed (KOH treatment-$\mathbf{A_{1r}}$) and Simulated Powder X-Ray Diffraction Patterns of A_1 (Note the peak due to eliminated KCl at 2θ=28º)

Table S3: Planes {h k l} passing through different 2θ values in $\mathbf{A_1}$

Entry No.	**2θ** (°)	***h k l***	Plane passing through
**1**	16.53	0 2 3	amine nitrogen
**2**	17.03	0 0 6	phenyl rings and parallel to *ab*-axis
**3**	18.70	1 2 2	amine nitrogen and bridged chlorine
**4**	19.74	1 2 3	amine nitrogen and bridged chlorine
**5**	20.19	1 0 6	benzyl carbon
**6**	26.93	0 2 8	amine nitrogen and parallel to two 1D Cu-Cl chain and on inversion centre
**7**	29	1 2 8	amine nitrogen, bridged chlorine and benzyl carbon
**8**	33.85	1 4 5	phenyl rings and Cu-N-Cu
**9**	34.62	3 0 4	Cu-N
**10**	38.60	1 5 3	Cu and amine group
**11**	49.36	2 6 2	amine nitrogen and bridged chlorine

4. Accommodative nature at Solid-Gas interface: Stereochemistry of Crystal to Crystal transformation

4.1. $\mathbf{A}_{S} \rightarrow \mathbf{B}_{S}$ crystal transformation.

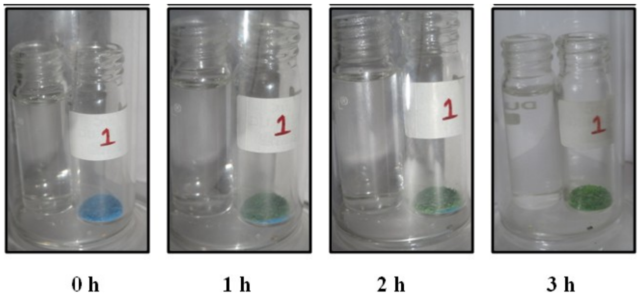

Figure S4: $\mathbf{A}_{S} \rightarrow \mathbf{B}_{S}$ transformation.

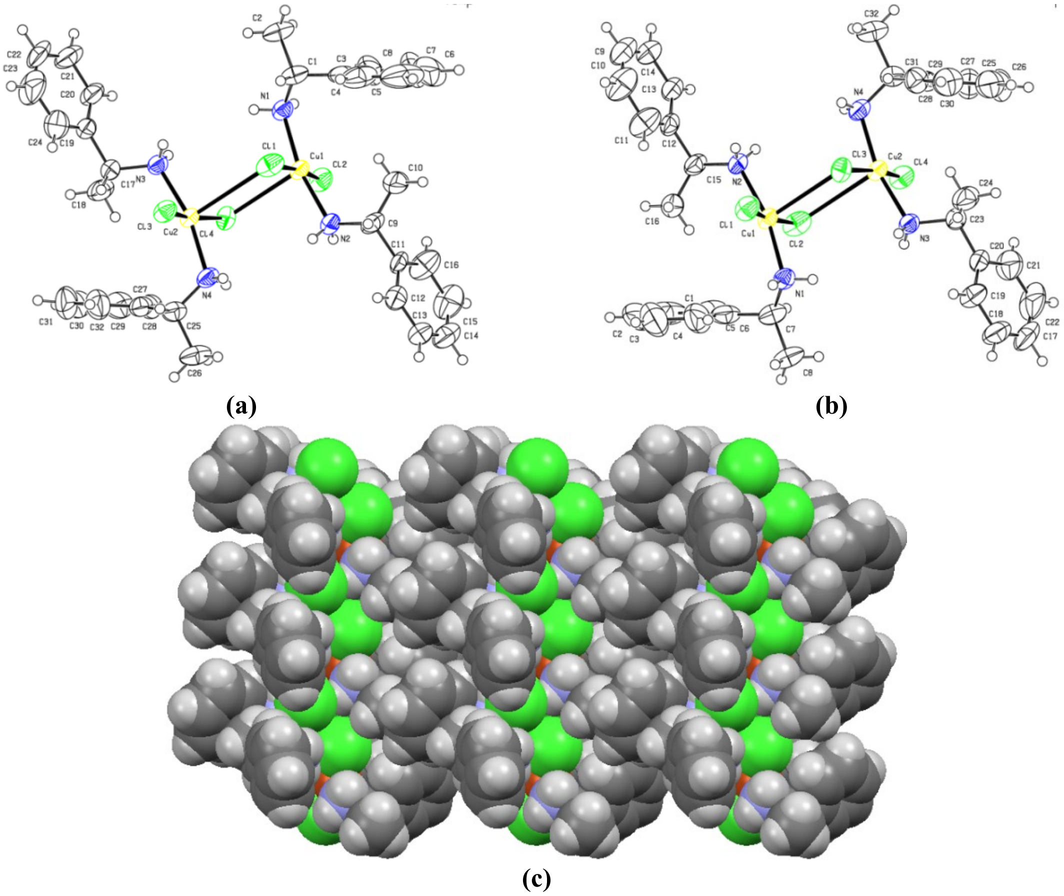

Figure S5: Molecular view of (a) $\mathbf{A}_{R}$ and (b) $\mathbf{A}_{S}$ (thermal ellipsoid are shown at 50 % probability); (c) $\mathbf{A}_{R}$ with space filled style denotes the absence of porosity.

4.2 X-ray crystal structure of B$_R$ and B$_S$ and X-Ray crystallography data

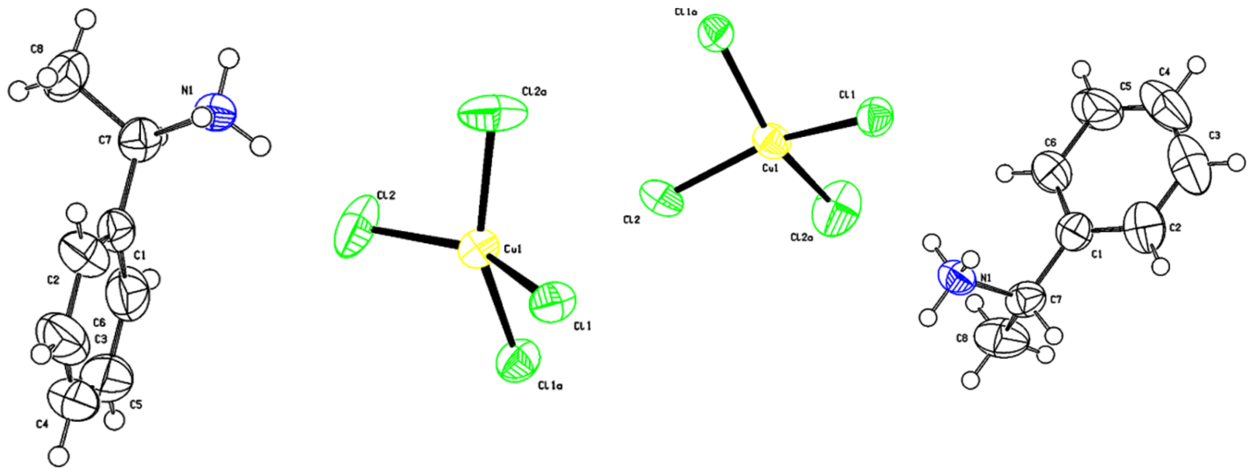

(b) **(c)**

Figure S6: Molecular view of **(**a) **B$_R$** and (b) **B$_S$** **(**thermal ellipsoid are shown at 50 % probability).

B$_R$ ((*R*)-(+)-Me-benzilinium)$_2$CuCl$_4$ and **B$_S$** ((*S*)-(-)-Me-benzilinium)$_2$CuCl$_4$ crystallized into monoclinic crystal system with C_2 space group. Here $[CuCl_4]^{2-}$ dianions are surrounded by organic ammonium cations and stabilized by hydrogen bonding interaction. Three N–H···Cl (3.180(2) Å) hydrogen bond and two bifurcated 2(N–H)···Cl (3.228(3) and 3.235(1)Å) hydrogen bonds were observed.

Table S4: Crystallographic data and structure refinements for s $\mathbf{B}_{\boldsymbol{R}}$ and $\mathbf{B}_{\boldsymbol{S}}$ at RT

	$\mathbf{B}_{\boldsymbol{R}}$	$\mathbf{B}_{\boldsymbol{S}}$
**CCDC**	**817150**	**817151**
Empirical formula	$C_{16}H_{24}Cl_4CuN_2$	$C_{16}H_{24}Cl_4CuN_2$
Formula weight	449.71	449.71
Wavelength (Å)	0.71073	0.71073
Crystal system	Monoclinic	Monoclinic
Space group	C_2	C_2
a (Å)	10.567(3)	10.582(3)
b (Å)	7.253(2)	7.257(2)
c (Å)	13.926(4)	13.940(3)
β(°)	95.99(3)	96.04(2)
V (Å3)	1061.5(5)	1064.5(5)
Z	2	2
Dcalc (Mg/m^3)	1.407	1.403
Reflections collected	2266	2263
Independent reflections	1848 [*R*int = 0.0185]	1688 [*R*int = 0.0209]
Goodness-of-fit on F^2	1.031	0.974
Data / restraints / Parameters	1848 / 1 / 105	1688 / 1 / 105
Final R indices [I>2α(I)]	R1 = 0.0324, wR2 = 0.0685	R1 = 0.0346, wR2 = 0.0633
R indices (all data)	R1 = 0.0404, wR2 = 0.0750	R1 = 0.0438, wR2 = 0.0689

4.4 Inner coordination and outer coordination sphere

Tables S5: Bond lengths of s A*R*, A*S*, B*R* and A*S*

	**A*R* (Å)**	**B*R* (Å)**	**A*S* (Å)**	**B*S* (Å)**
**Cu(1)-Cl(1)**	2.295(17)	2.264(9)	2.292(16)	2.269(11)
**Cu(1)-Cl(1#)**	2.317(17)	2.264(9)	2.319(16)	2.269(11)
**Cu(1)-Cl(2)**		2.239(11)		2.239(12)
**Cu(1)-Cl(2#)**		2.239(11)		2.239(12)
**Cu(1)-Cl(3)**	2.828(2)		2.836(2)	
**Cu(2)-Cl(2)**	2.285(16)		2.286(16)	
**Cu(2)-Cl(2#)**	2.285(17)		2.282(15)	
**Cu (1)-N(1)**	2.001(5)		2.015(3)	
**Cu (1)-N(2)**	2.010(5)		1.997(3)	

4.5 Solid state absorption spectra:

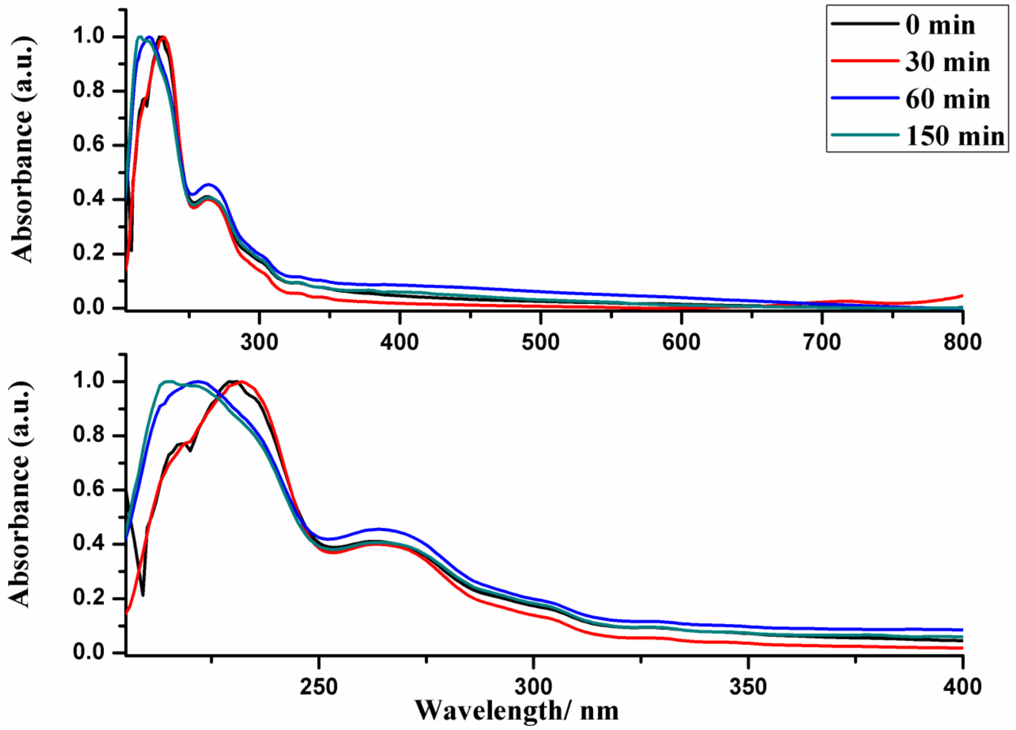

Figure S7: Solid state absorption spectra of conversion of $\mathbf{A}_S \rightarrow \mathbf{B}_S$

The electronic spectra of $\mathbf{A}_S$ showed the absorbance maxima at 230nm as shown in Fig.S7. It does not showed any sign of d-d transition. After 30 min. exposure with HCl, d-d transitions band centered around 717nm observed a five- coordinate copper(II) environment.

The electronic spectra of copper (II) complexes with square-pyramidal geometries have been studied extensively by Hathaway and coworkers [1]. It was possible to predict the stereochemistry of the local copper (II) ion environment in five-coordinated complexes from the positions and intensities of the d-d transition bands of CuX5 chromophores. The electronic spectra of distorted square-based pyramidal complexes consist of a single or two bands covering the range 667 ±50nm [2]. This distortion is towards a tetrahedral arrangement of the basal plane. The position of the d-d band in $\mathbf{A}_S$ is very similar to that reported for the copper(II) complexes in which copper(II) atoms have a distorted tetrahedral-based pyramidal geometry suggesting the addition of chlorine atom to copper metal.

After 60 minutes exposure to HCl vapours, showed hypsochromic and hypochromic shift in the spectra. The spectra of complex showed strong band around 221nm suggesting that formation of

$\mathbf{B}_S$. No sign of d-d transition was observed at this stage. At 150 min. more hypsochromic shift 214nm in the spectra confirms the formation $\mathbf{B}_S$ from $\mathbf{A}_S$.

[1] (a) H.-L. Zhu, P. Huang, C.-Y. Duan, L.-M. Zheng, Y.-J. Liu, M.-F. Wu, W.-X. Tang, *Transition Met. Chem.* **1999**, *24*, 380-383; (b) B. J. Hathaway, A. A. G. Tomlinson, *Coord. Chem. Rev.* **1970**, *5*, 1-43; (c) B. J. Hathaway, *J. Chem. Soc., Dalton Trans.* **1972**, 1196-1199.

[2] P. Chaudhuri, K. Oder, *J. Chem. Soc., Dalton Trans.* **1990**, 1597-1605.

4.6. *ex-situ* FT-IR

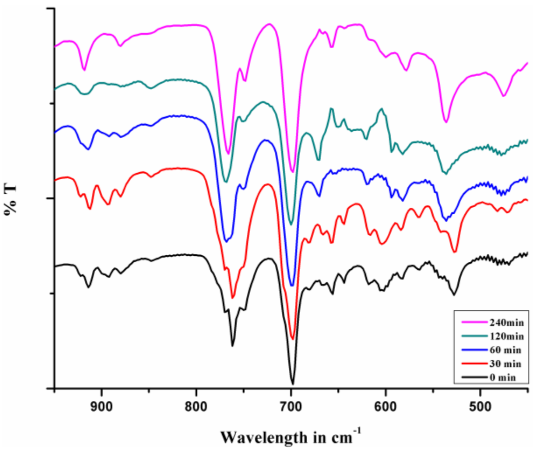

(a)

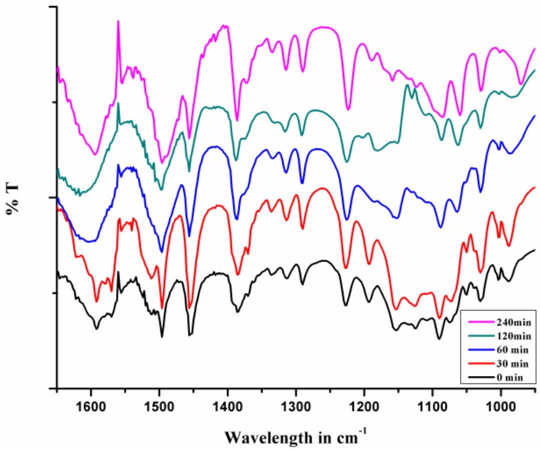

(b)

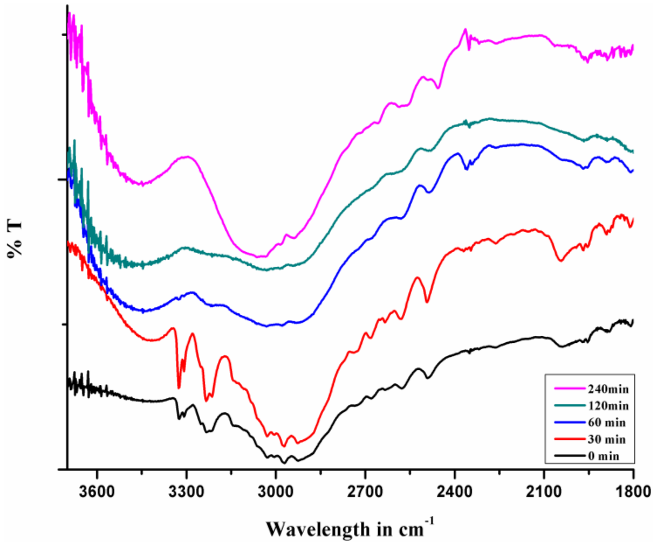

(c)

Figure S8: (a-c) *ex-situ* FT-IR monitoring of $\mathbf{A}_R/\mathbf{A}_S \rightarrow \mathbf{B}_R/\mathbf{B}_S$ in KBr pellet.

The FT-IR spectrum of $\mathbf{A}_{\boldsymbol{R}}$/$\mathbf{A}_{\boldsymbol{S}}$ was recorded in solid state. The KBr pellet (3mg of $\mathbf{A}_{\boldsymbol{R}}$/$\mathbf{A}_{\boldsymbol{S}}$ in 100mg KBr) of $\mathbf{A}_{\boldsymbol{R}}$/$\mathbf{A}_{\boldsymbol{S}}$ were kept in HCl gas environment and FT-IR spectra were recorded with increase of exposure time.

After 30 min. almost all bands retain their position but color of pellet changes from green to green yellow. After one hour substantial change occurred and pellet becomes brown. IR spectra indicate the loss of -NH_2 symmetric and antisymmetric stretching vibration and formation of -NH_3^+ stretching vibration at 3028-3055cm^{-1}. Formation of weak band at 1622cm^{-1} indicates the -NH_3^+ symmetric bending mode. -CH_3 deformation mode became a sharp while -CH_2 rocking mode merged in single band and shifted slightly. Out of plane ring deformation modes get merged and became sharp. After 60 mins all bands become sharp in nature without further change up to 120 mins. After 240 mins $\mathbf{A}_{\boldsymbol{R}}$/$\mathbf{A}_{\boldsymbol{S}}$ transforms to $\mathbf{B}_{\boldsymbol{R}}$/$\mathbf{B}_{\boldsymbol{S}}$. (Comparison with standard IR spectrum of $\mathbf{B}_{\boldsymbol{R}}$/$\mathbf{B}_{\boldsymbol{S}}$).

4.7 Powder XRD

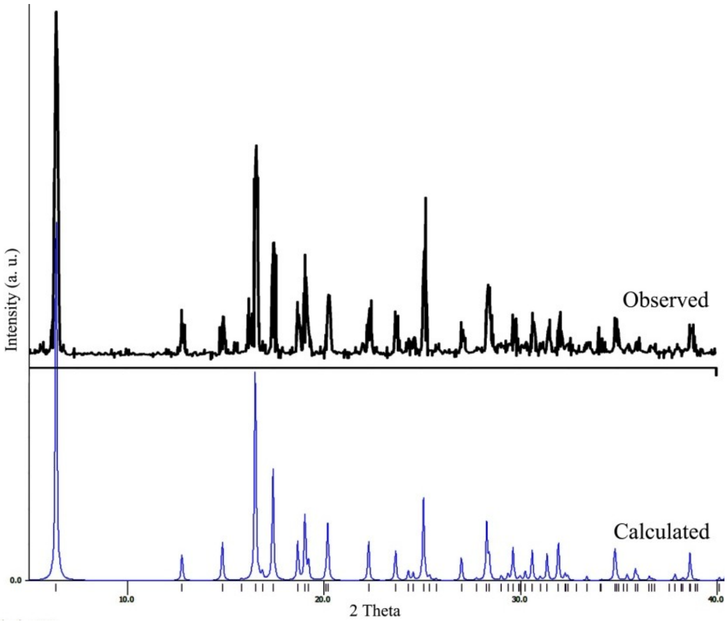

(a)

Figure S9: PXRD pattern of $\mathbf{B}_{R}$ obtained after keeping $\mathbf{A}_{R}$ during 150 minutes in the HCl gas environment after the solid-gas interface synthesis. Wavelength: 1.54056 Å.

10.2 *ex-situ* reaction monitoring

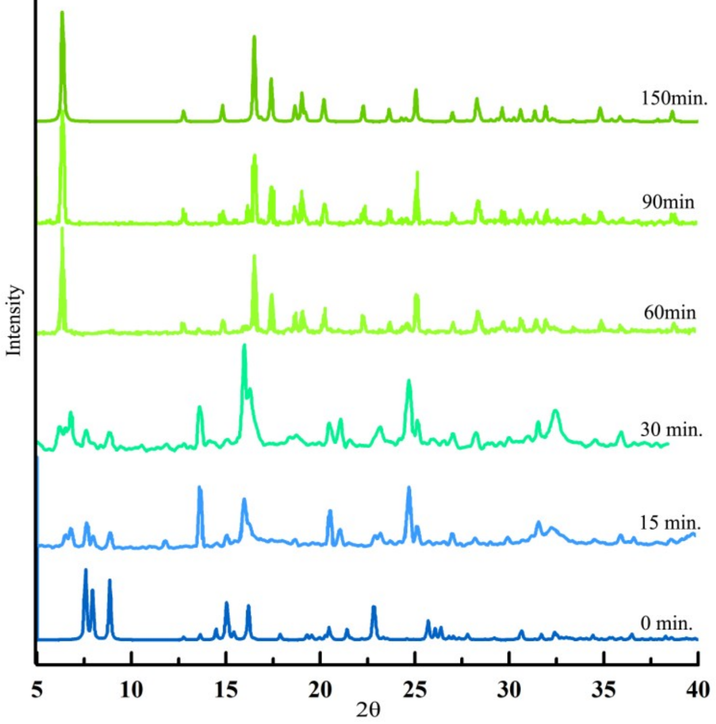

(b)

Figure S10: PXRD time-dependent transformation of $\mathbf{A}_{R}$ to $\mathbf{B}_{R}$ in HCl gas environment measured at 298 K. Wavelength: 1.54056Å.

4.7.3 $B_S \rightarrow A_S$ after KOH treatment

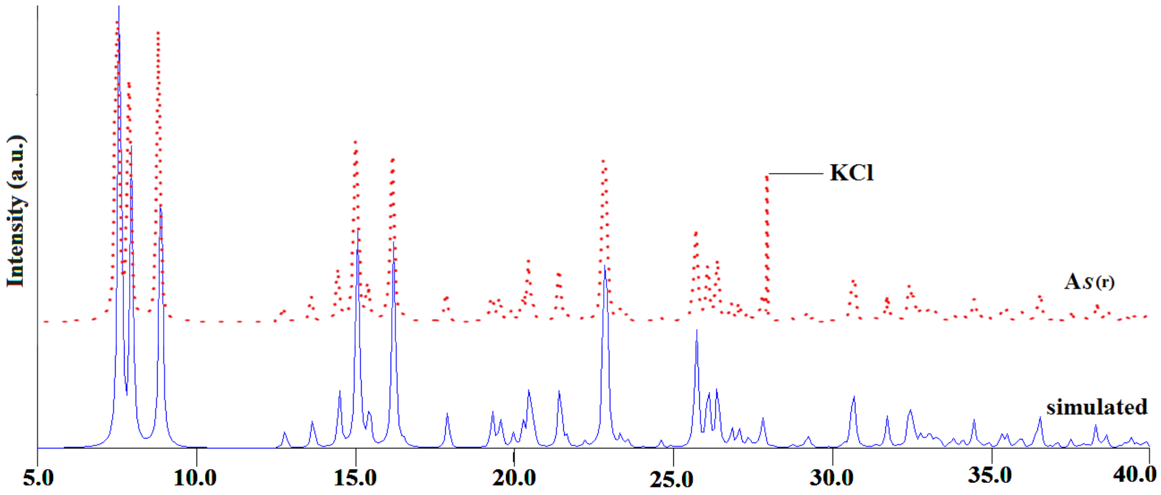

(c)

Figure S11: XRPD of transformation of $\mathbf{B}_S \rightarrow \mathbf{A}_S$ **(r)** after KOH. (Note the peak due to eliminated KCl at 2θ=28°)

4.8 Solid state CD spectra

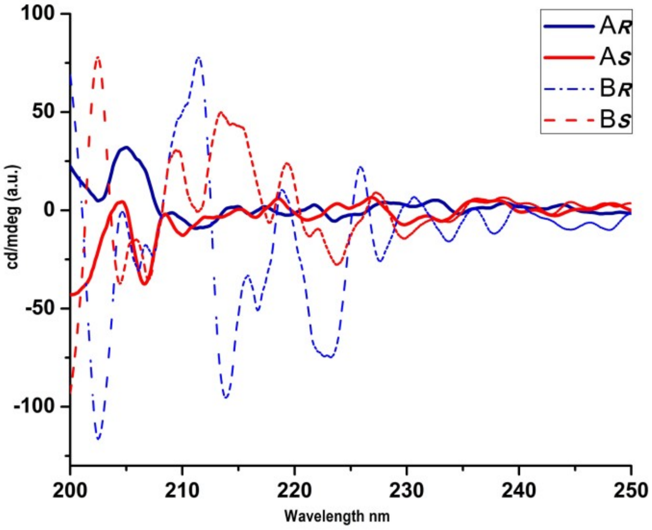

Figure S12: Solid state CD spectra of $\mathbf{A}_{\boldsymbol{R}}$, $\mathbf{A}_{\boldsymbol{S}}$, $\mathbf{B}_{\boldsymbol{R}}$ and $\mathbf{B}_{\boldsymbol{S}}$ in nujol.

$\mathbf{A}_{\boldsymbol{R}}$ exhibits a negative dichroic signal at 204 nm and positive dichroic signal at 206 nm while $\mathbf{A}_{\boldsymbol{S}}$ shows Cotton effect with opposite sign at the same wavelength.

$\mathbf{A}_{\boldsymbol{S}}$ exhibits a positive dichroic signal at 205 nm which shifted to blue region at 202 nm during the transformation along with generation of new dichroic signals. After conversion, $\mathbf{B}_{\boldsymbol{R}}$ exhibits a negative dichroic signal at 203, 214 and 224 nm and a positive Cotton effect at 212 and 220 nm. $\mathbf{B}_{\boldsymbol{S}}$ shows Cotton effects of the opposite sign at the same wavelengths.

Grinding $\mathbf{B}_{\boldsymbol{S}}$ with KOH led to a color change from yellow green into bluish green in minutes. No change in sign of cotton effect during conversion of $\mathbf{B}_{\boldsymbol{S}}$ to $\mathbf{A}_{\boldsymbol{S}}$ **(r)**.

4.9 Specific Optical Rotation (SOR) activity on A_R and A_S, B_R and B_S

TableS6: SOR data on $\mathbf{A_R}$ and $\mathbf{A_S}$, $\mathbf{B_R}$ and $\mathbf{B_S}$ in methanol at 30ºC.

Sr. No.		Angle of minimum deviation (°)			Average (°)
1	$\mathbf{A_R}$	+40.8	+38.4	+37.6	+38.9º
2	$\mathbf{A_S}$	-33.8º	-33.0º	-38.4º	-35.1º
3	$\mathbf{B_R}$	-7.9º	-7.7º	-8.4º	-8.0º
4	$\mathbf{B_S}$	-7.2º	-7.7º	-8.6º	-7.9º

12.2 *ex-situ* reaction monitoring of conversion of A_S to B_S and $B_S \rightarrow A_S$ with time of exposure to HCl

TableS7: *ex-situ* reaction monitoring with time of conversion of $\mathbf{A_S}$ to $\mathbf{B_S}$ using **SOR** in methanol and S1(r) showed SOR of conversion of $\mathbf{B_S} \rightarrow \mathbf{A_S}$ at **30ºC**.

Sr. No.	$A_S \rightarrow B_S$ with time	Angle of minimum deviation (°)			Average (°)
1	0 min	-38.4º	-39.3	-38.4º	-38.4º
2	60 min	-7.1º	-7.3º	-7.6º	-7.4º
3	150 min	-8.2º	-7.7º	-8.5º	-8.1º
4	S1(r)*	-21.07	-21.09	-21.2	-21.1

* After KOH treatment.

$\mathbf{A_R}$ has a specific rotation of +38.4º while enantiomer $\mathbf{A_S}$ has a specific rotation of -38.4º at 30ºC (Table S6). $\mathbf{A_S}$ after 60 minutes exposure to HCl vapors became bluish green with specific rotation of -7.4º. The reason behind decline in SOR value may be due to formation of ammonium cation of (*S*)-(−)-α-Methyl benzylamine ligand. After 150 min., $\mathbf{A_S}$ completely transformed to $\mathbf{B_S}$ with specific rotation of -8.1º which completely matches with the standard SOR activity (-7.9º) of $\mathbf{B_S}$.

Grinding $\mathbf{B_S}$ with KOH results into bluish green powder from yellow green, with SOR activity -21.1º. Decrease in SOR value may be due to presence of water along with methanol. This study

suggests that during inter conversion of **A*S*** → **B*S*** and reversibly from **B*S*** → **A*S*** happens with no change in the absolute configuration of ligand, no racemisation during transformation.

4.10 Theoretical study

$\mathbf{A}_{S}$

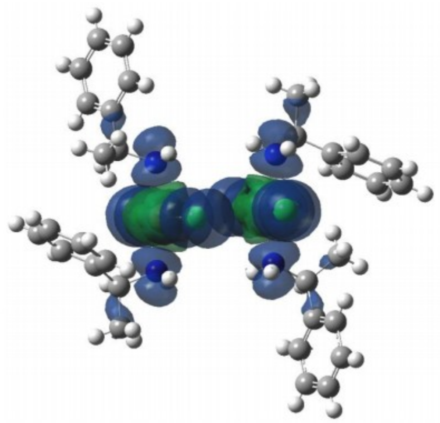

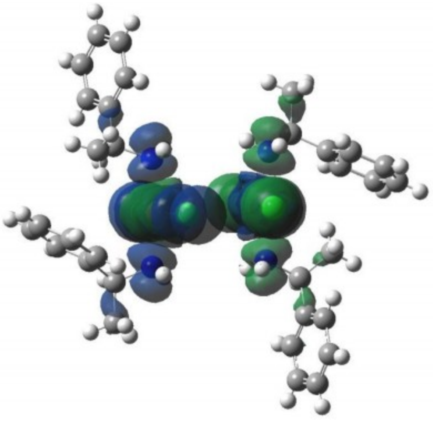

Triplet State	Singlet State
Spin density = 2.0	Spin density = 0.0

$\mathbf{B}_{S}$

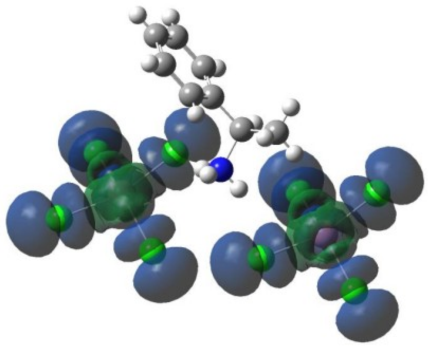

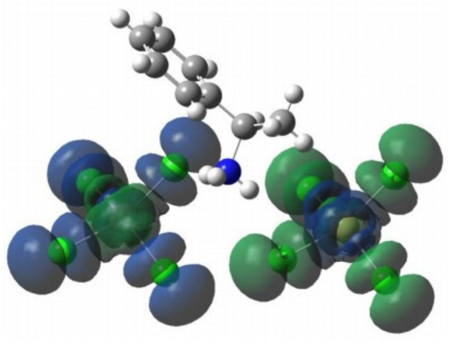

Triplet State	Singlet State
Spin density = 2.0	Spin density = 0.0

For both complexes ΔE = E(Singlet) – E(Triplet) ≈ 0

Figure S13: Theoretical study on $\mathbf{A}_{S}$ and $\mathbf{B}_{S}$

Theoretical study on s $\mathbf{A}_{S}$ and $\mathbf{B}_{S}$ were carried out using UB3LYP/6-31+G(d,p) method. LANL2DZ basis set for Cu atom. $\mathbf{A}_{S}$ showed that the spin density in triplet state resides mainly on Cu and Cl atoms, partially on the NH_2 groups while it is zero in singlet state. $\mathbf{B}_{S}$ shows the spin density in triplet state resides only on Cu and Cl atoms and not on the ligand while it is zero in singlet state.

4.11 Different structures of ligands

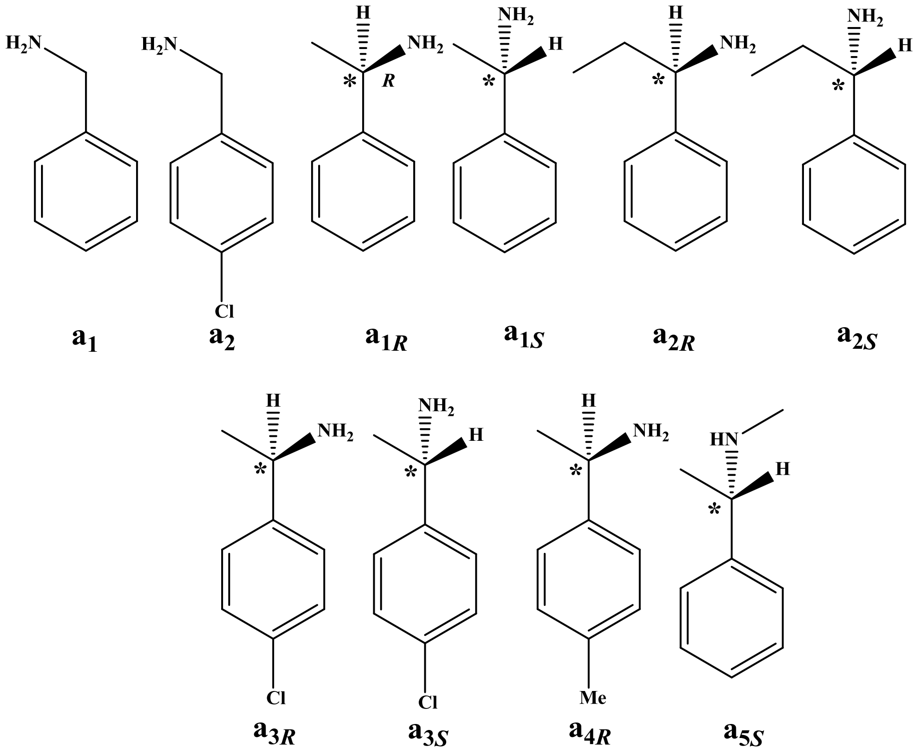

Figure S14: Different structures of ligands employed during present investigation.

4.12 Solid state EPR

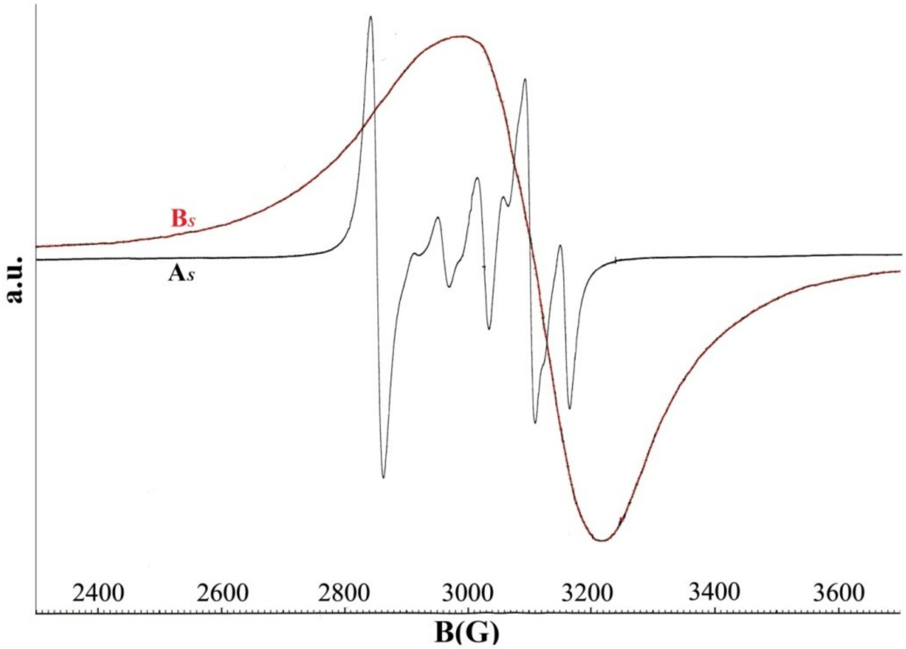

Figure S15: Solid state EPR spectra of **A*S*** and **B*S*** at room temperature.

A*S*

g||= 2.14; g⊥ = 2.13; $g_{avg.}$= 2.13

B*S*

g||= 2.16; g⊥ = 2.08; gavg.= 2.13

4.13 DSC and Thermochromism

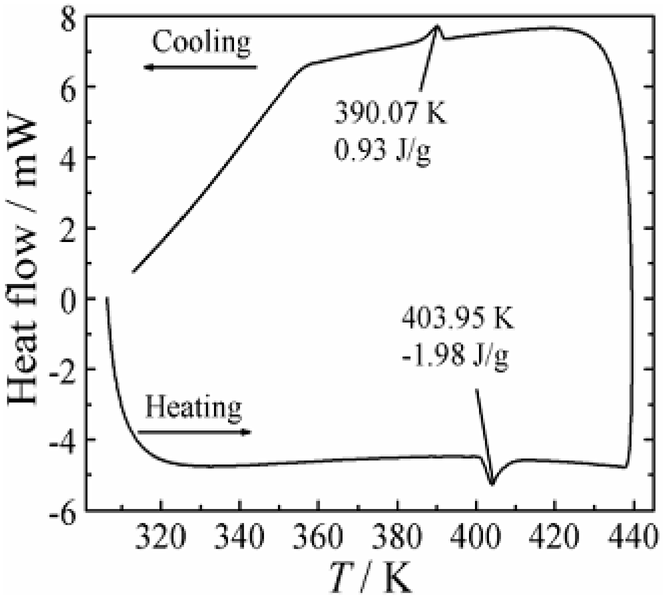

(a)

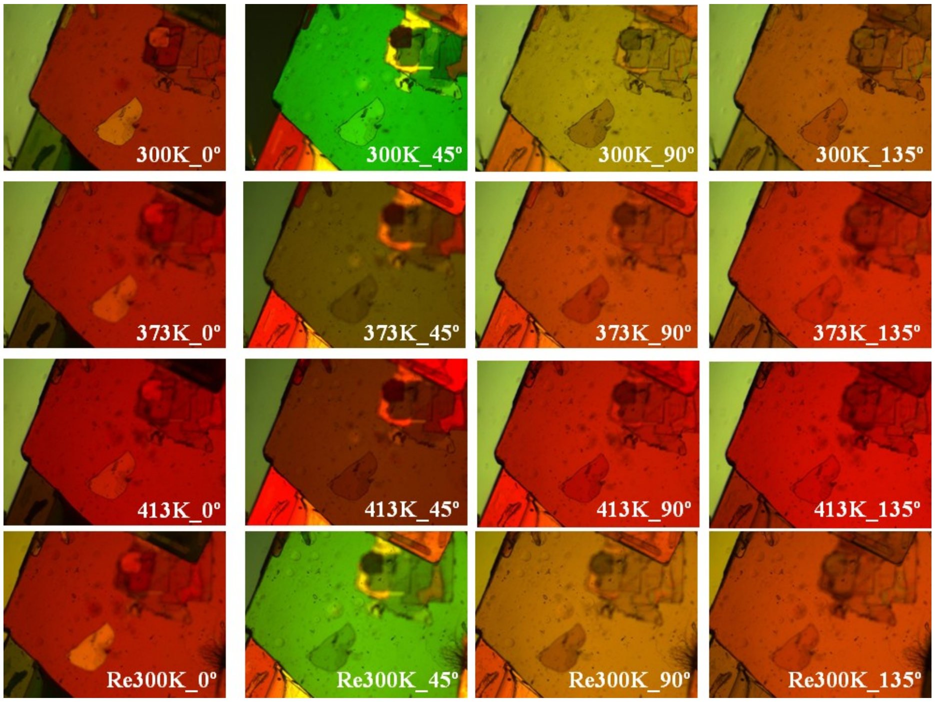

(b)

Figure S16: (a) DSC and **(b)** Thermochromic behavior for $\mathbf{B_1}$ using polarizing microscope.

4.14 DSC and Thermochromic behavior for B_R / B_S

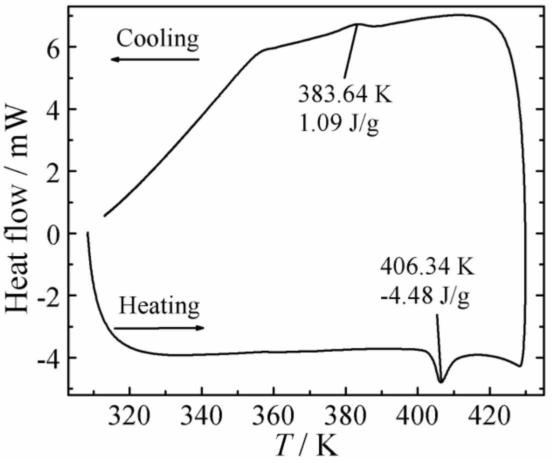

(a)

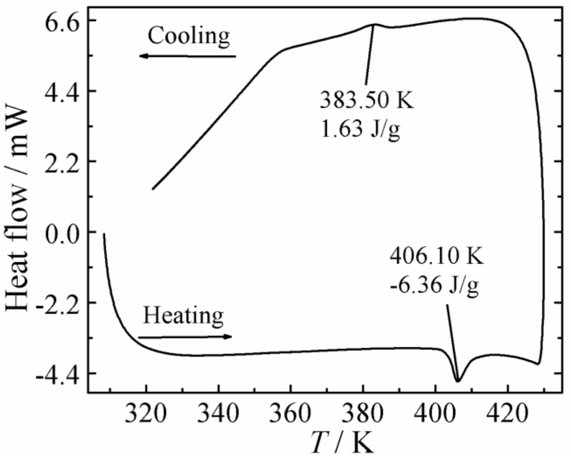

(b)

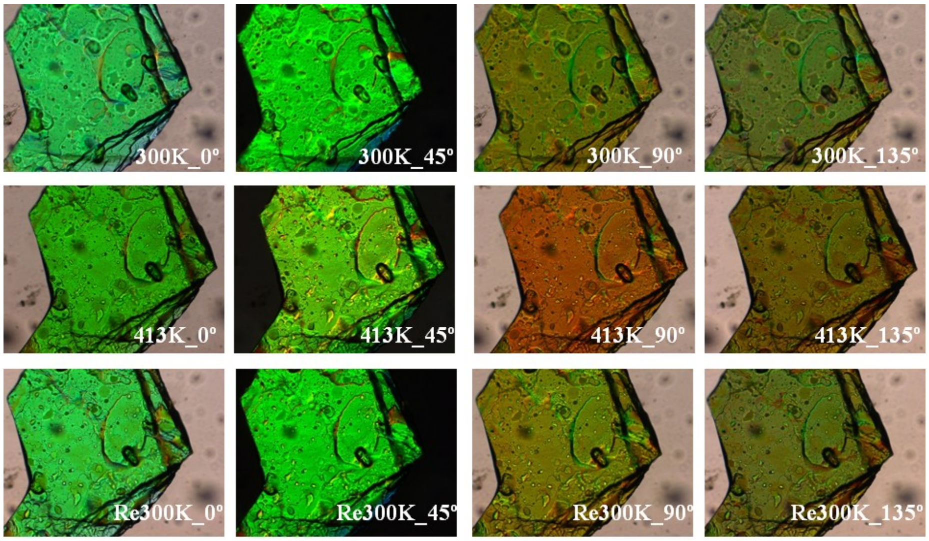

Figure S17: (a) DSC of $\mathbf{B}_R$, (b) DSC of $\mathbf{B}_S$ and (c) Thermochromic behavior for $\mathbf{B}_R$ / $\mathbf{B}_S$ using polarizing microscope.

4.15. PE Loop measurement

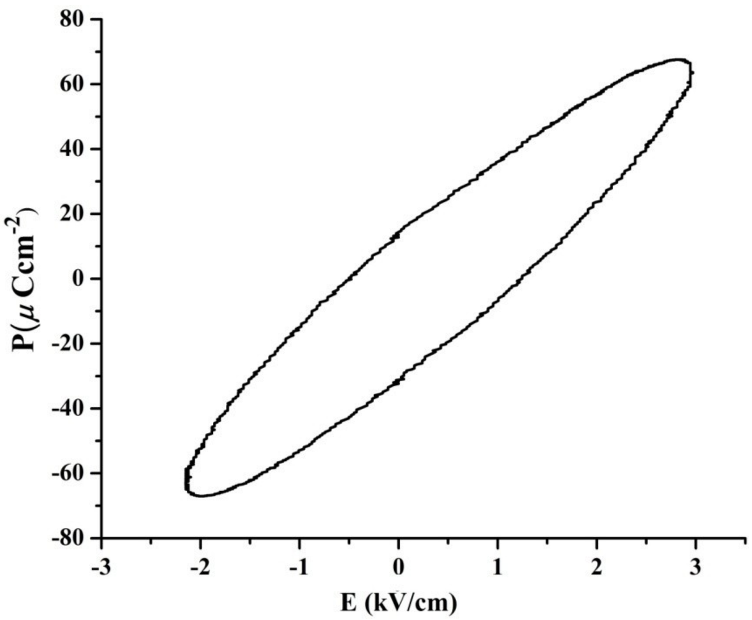

Figure S18: PE Loop measurement of $\mathbf{B}_{\boldsymbol{R}}$

A ferroelectricity loop measurements was performed on $\mathbf{B}_{\boldsymbol{R}}$ in pellet. At 300 K, we observed large spontaneous polarization, switchable with a relatively small electric field at room temperature. The ferroelectricity loop measurements (PE loop) on $\mathbf{B}_{\boldsymbol{R}}$ is depicted in Fig.S18.

Hysteresis measurements gave a well-defined loop for $\mathbf{B}_{\boldsymbol{R}}$ with the maximum field of 0.865kVcm^{-1}. The coercive field Ec observed at 0.865 kVcm^{-1} while remnant polarization *P*r observed at 155.10 μCcm^{-2} at 300 K.

5. Crystal to Crystal transformation at Solid-Liquid interface: Self assembly, Molecular Recognition and Anchimeric assistance

5.1 X-ray crystal structure of C_R, C_S and its crystal packing

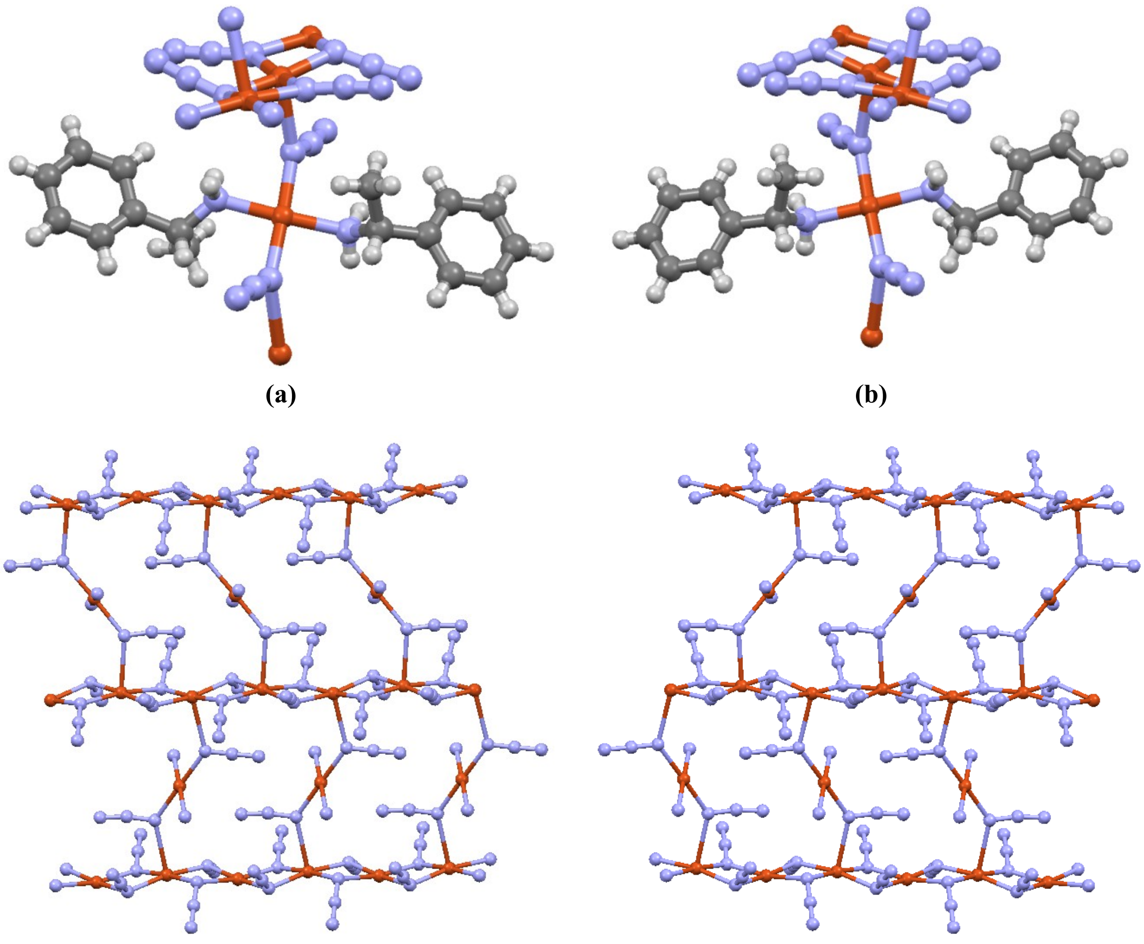

(a) (b) (c) (d)

Figure S19: Molecular view of (a) $\mathbf{C_R}$ and (b) $\mathbf{C_S}$; Arrangement of the 2D brick-wall network (c) $\mathbf{C_R}$ and (d) $\mathbf{C_S}$ (H and C atoms of the amine ligands are omitted for clarity)

5.2 Powder XRD

5.2.1 Comparison of observed XRPD pattern of bulk sample and simulated XRPD pattern (from single crystal data)

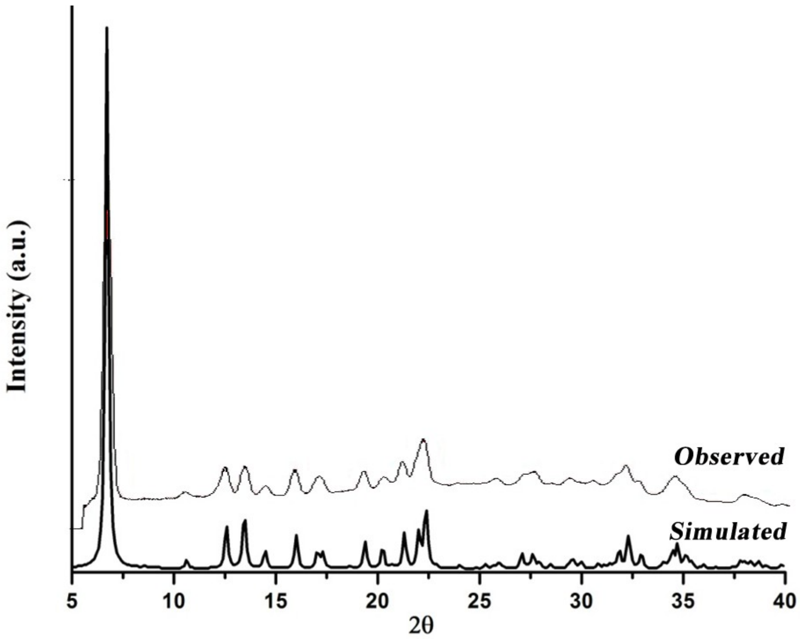

Figure S20: Observed and simulated XRPD pattern of $\mathbf{C}_{\boldsymbol{R}}$ (obtained from $\mathbf{A}_{\boldsymbol{R}}$)

5.2.2 Comparison of observed XRPD pattern of bulk sample and simulated XRPD pattern (from single crystal data)

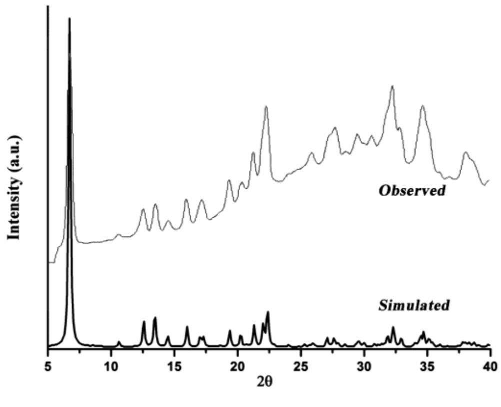

(a)

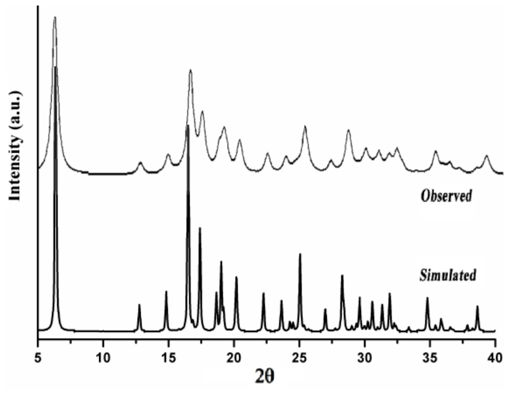

(b)

Figure S21: (a) Observed and simulated XRPD pattern of $\mathbf{C}_{\boldsymbol{R}}$ (obtained from $\mathbf{B}_{\boldsymbol{R}}$); (b) Observed and simulated XRPD pattern of $\mathbf{B}_{\boldsymbol{R}}$ (obtained from $\mathbf{C}_{\boldsymbol{R}}$).

5.3 Solid state CD spectra

5.3.1 Solid CD spectra of A_R, A_S, C_R and C_S

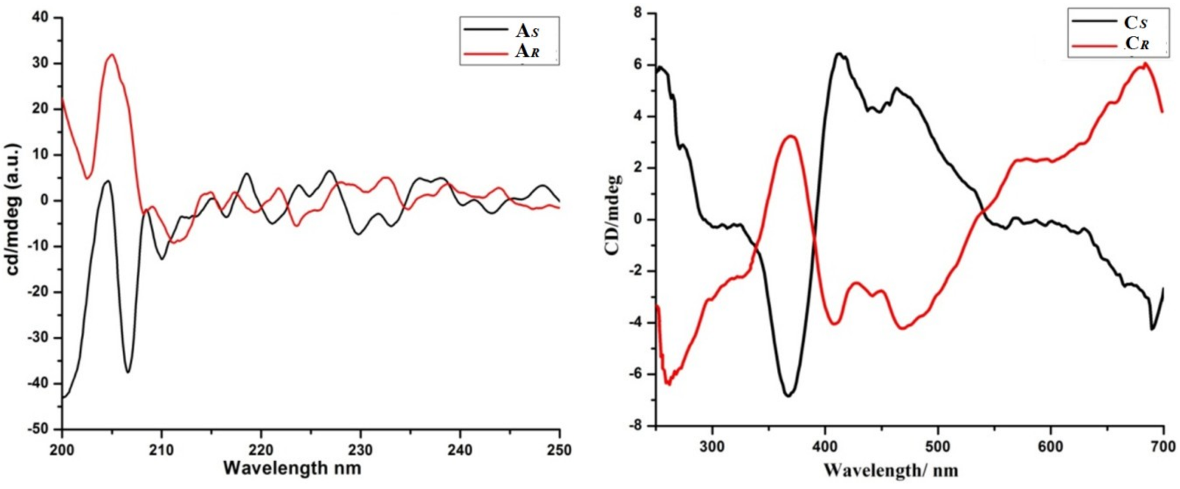

(a) (b)

Figure S22: Solid state CD spectra in nujol (a) $\mathbf{A_R}$ and $\mathbf{A_S}$; (b) $\mathbf{C_R}$ and $\mathbf{C_S}$.

5.3.2 Solid CD spectra of B_R, B_S, C_R and C_S

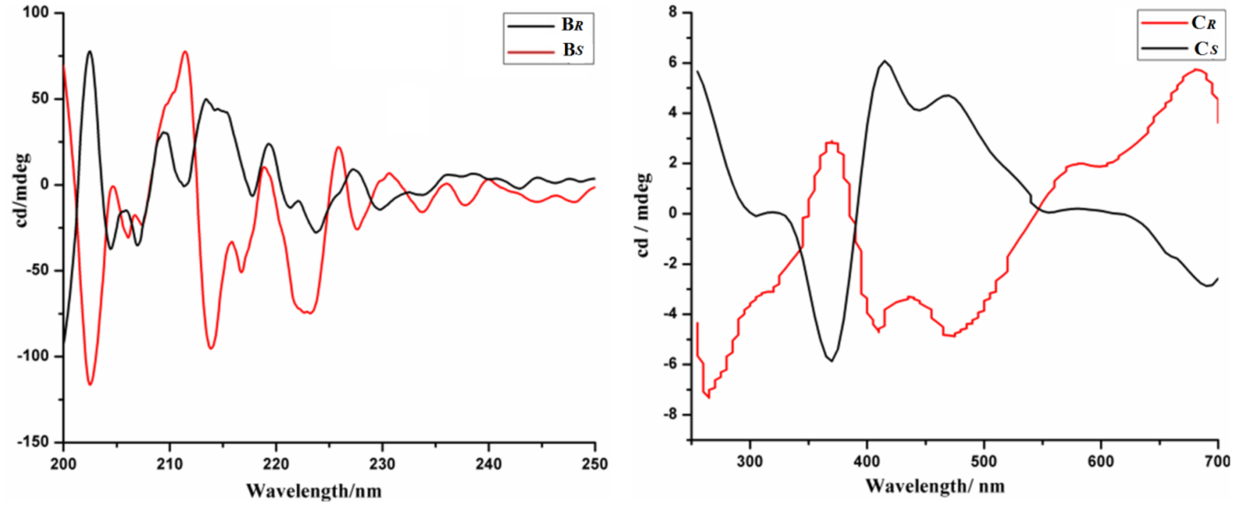

(a) (b)

Figure S23: Solid state CD spectra in nujol (a) $\mathbf{B_R}$ and $\mathbf{B_S}$; (b) $\mathbf{C_R}$ and $\mathbf{C_S}$.

6. Indexed Powder XRD

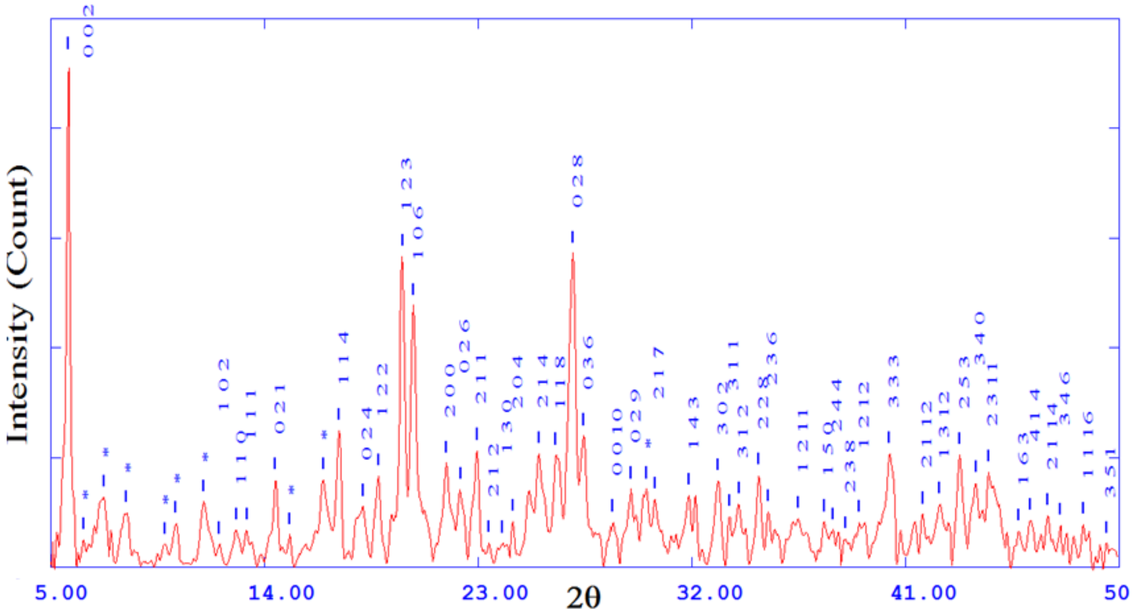

A$_1$

Crystal system: Orthorhombic; **Lattice Type**: P; **Radiation**: Cu; **Wavelength**: 1.54178; **Lattice Parameter**: a= 8.2260 b= 12.4900 c= 31.207; **Lattice Parameter**: α= 90, β= 90, γ=90

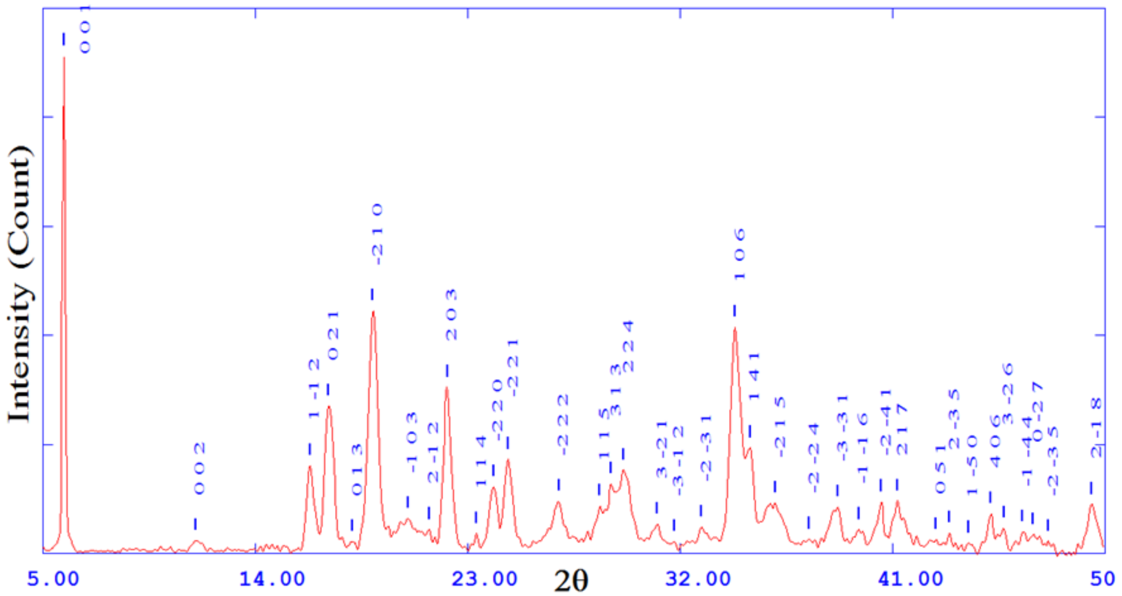

B$_1$

Crystal system: Triclinic; **Lattice Type**: P; **Radiation**: Cu; **Wavelength**: 1.54178; **Lattice Parameter**: a= 10.55 b= 10.55 c= 15.9114; **Lattice Parameter**: α = 81.218 β = 80.399 γ =89.237

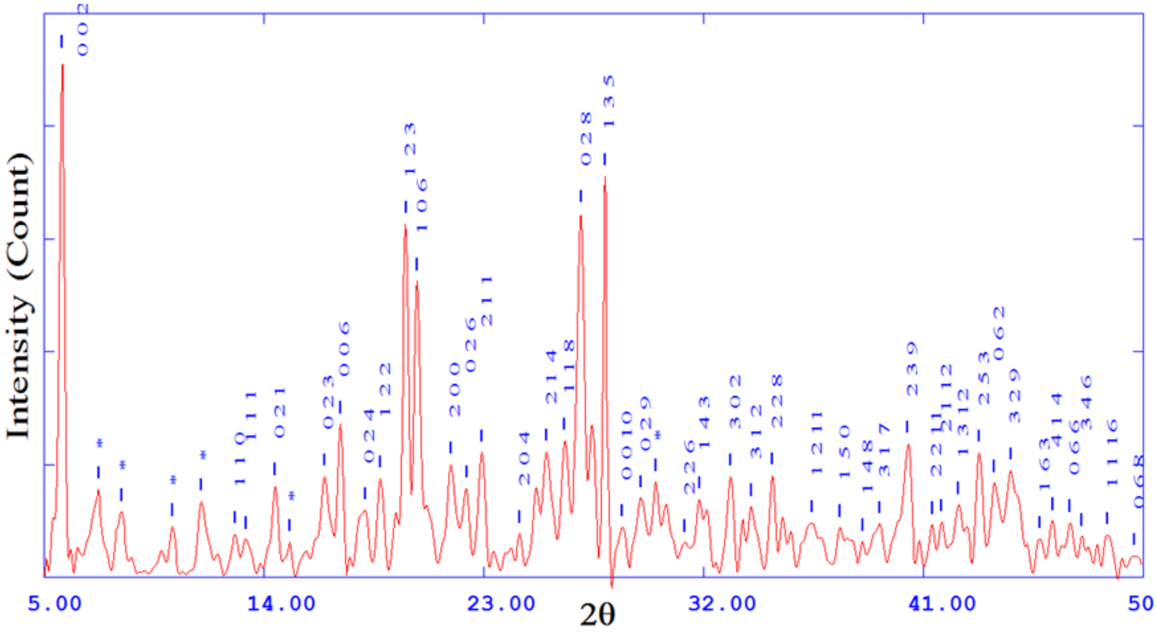

A$_1$ (r)

Crystal system: Orthorhombic; **Lattice Type**: P; **Radiation**: Cu; **Wavelength**: 1.54178; **Lattice Parameter**: a= 8.2320 b= 12.510 c= 31.219; **Lattice Parameter**: α= 90, β= 90, γ=90

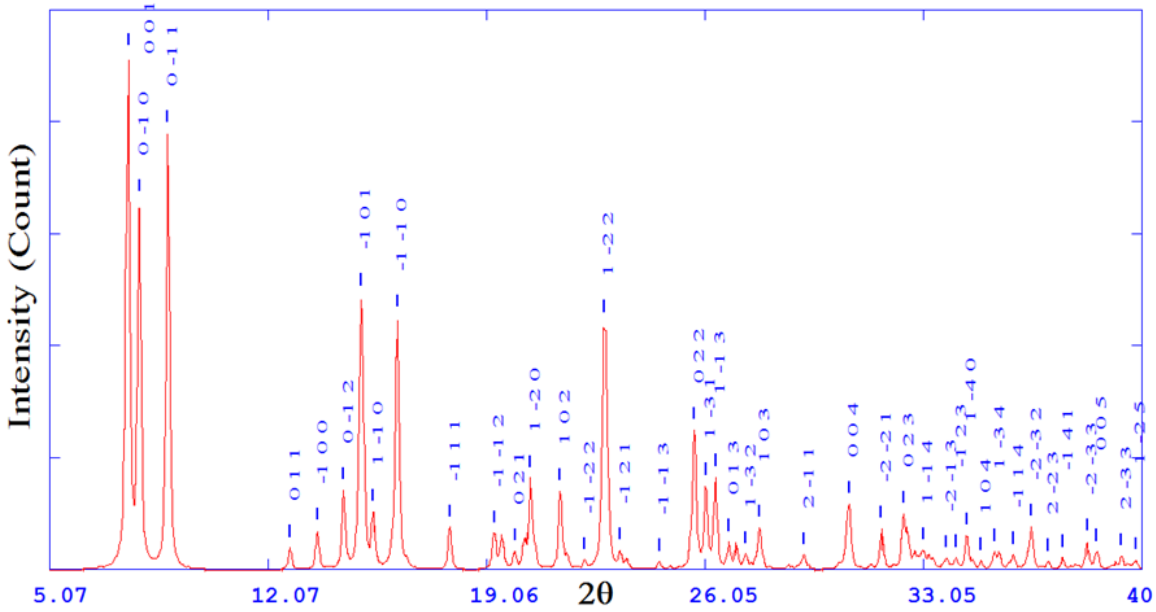

A$_R$

Crystal system: Triclinic; **Lattice Type**: P; **Radiation**: Cu; **Wavelength**: 1.54178; **Lattice Parameter**: a= 6.5134 b= 11.8561 c= 12.4718; **Lattice Parameter**: α = 110.209 β = 94.371 γ =91.485

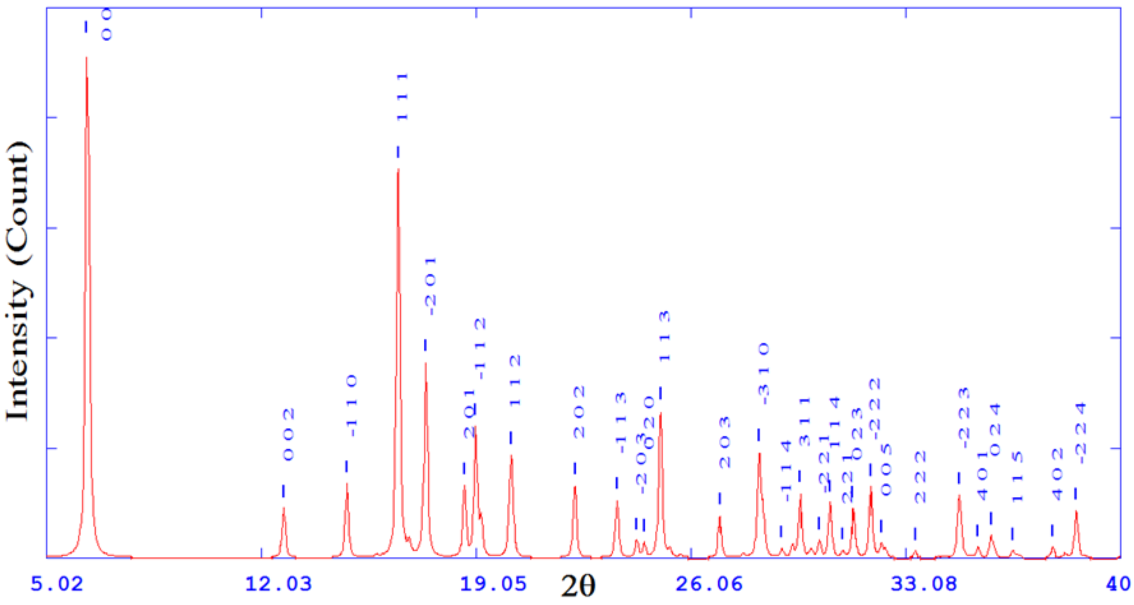

B$_R$

Crystal system: Monoclinic; **Lattice Type**: P; **Radiation**: Cu; **Wavelength**: 1.54178; **Lattice Parameter**: a= 10.5669 b= 7.251 c= 13.9263; **Lattice Parameter**: α= 90, β= 95.991, γ=90

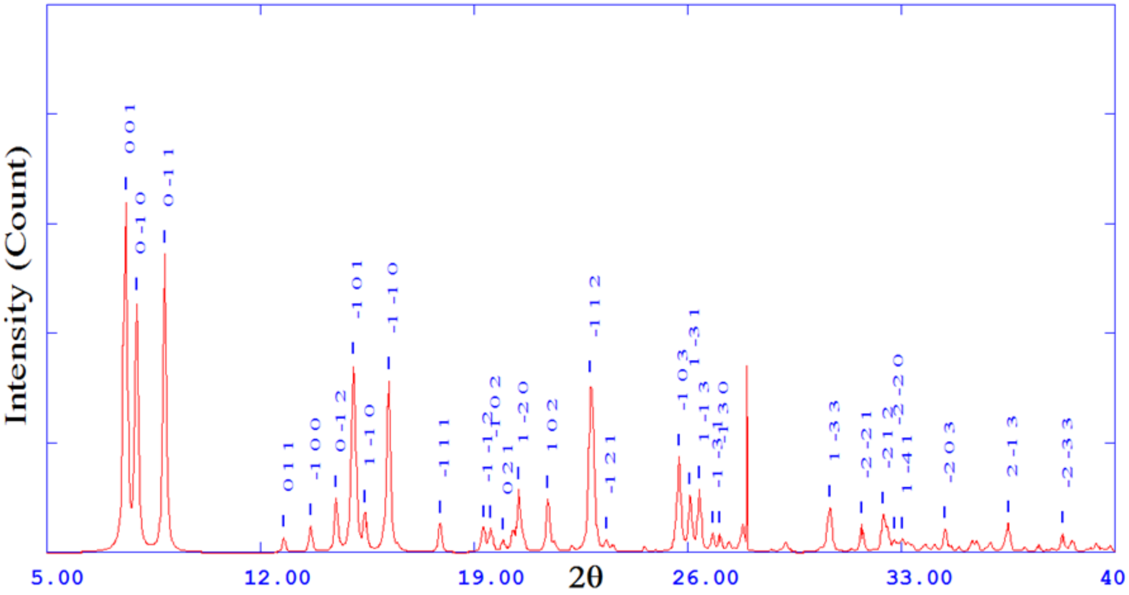

A$_R$(r)

Crystal system: Triclinic; **Lattice Type**: P; **Radiation**: Cu; **Wavelength**: 1.54178; **Lattice Parameter**: a= 6.5054 b= 11.8452 c= 12.4608; **Lattice Parameter**: α = 110.209 β = 94.371 γ =91.485
